# Treatment of pediatric flatfoot: a systematic review-based consensus and guidelines by CPAM-LRC

**DOI:** 10.3389/fped.2026.1825355

**Published:** 2026-05-08

**Authors:** Sharula, Fei Zhao, Tianyi Wu, Qinglin Kang, Xiang Geng, Hui Qin, Yan Zhang, Jin He, Lihua Huang, Jia Xu, Shengdi Lu

**Affiliations:** 1 Department of Orthopedics, Shanghai Sixth People’s Hospital Affiliated to Shanghai Jiao Tong University School of Medicine, Shanghai, China; 2 Department of Orthopedics, Taihe Hospital Affiliated to Hubei University of Medicine, Shiyan, Hubei, China; 3 Department of Foot and Ankle Surgery, Shanghai Sixth People’s Hospital Affiliated to Shanghai Jiao Tong University School of Medicine, Shanghai, China; 4 Department of Foot and Ankle Surgery, Huashan Hospital, Fudan University, Shanghai, China; 5 Department of Pediatric Orthopedics, Shanghai Sixth People’s Hospital Affiliated to Shanghai Jiao Tong University School of Medicine, Shanghai, China; 6 Department of Orthopedics, Xinhua Hospital Affiliated to Shanghai Jiao Tong University School of Medicine, Shanghai, China; 7 Department of Rehabilitation, Shanghai Sixth People’s Hospital Affiliated to Shanghai Jiao Tong University School of Medicine, Shanghai, China; 8 Chronic Disease Epidemiology Laboratory, Pennington Biomedical Research Center, Baton Rouge, LA, United States

**Keywords:** calcaneal osteotomy, clinical practice guideline, delphi consensus, flexible pes planus, foot orthoses, pediatric flatfoot, subtalar arthroereisis

## Abstract

The purpose of this clinical practice guideline is to provide evidence-based recommendations for the treatment of pediatric flexible flatfoot, developed in accordance with the Appraisal of Guidelines for Research and Evaluation II framework and with evidence certainty assessed using the GRADE framework and the Oxford Centre for Evidence-Based Medicine levels of evidence system. A multidisciplinary guideline development group under the Limb Reconstruction Committee of the Orthopedics Branch of China International Exchange and Promotion Association for Medical and Health Care systematically searched and reviewed evidence from primary studies including randomized controlled trials, cohort studies, and comparative studies, supplemented by existing systematic reviews and expert society surveys, to evaluate the effectiveness of conservative and surgical interventions and to guide clinicians and families on the content of an optimal treatment pathway. The guideline targets children and teenagers with flexible flatfoot and addresses interventions available to orthopedic surgeons, podiatrists, rehabilitation physicians, and orthotists, including observation, rehabilitative exercises, foot orthoses, subtalar arthroereisis, calcaneal osteotomy, and criterion-based progression to surgery. Structured conservative management should be considered the mainstay of care for all symptomatic children, with a minimum 6-month trial before surgical referral. However, there is limited evidence on the optimal type, dose, and duration of conservative treatment, and what constitutes an adequate trial of nonoperative care remains undefined. Foot orthoses can be helpful for symptomatic relief when pain or functional limitation is present, and rehabilitative exercise programs may allow superior normalization rates compared to orthoses alone. Pain-free ambulation and return to unrestricted sport are key milestones for both conservative and surgical pathways. However, no validated progression or discharge criteria exist to guide the transition from one treatment phase to the next. While the certainty of evidence was low to very low for most components of the treatment pathway, all 15 recommendation statements were formulated through two rounds of Delphi consensus polling, with 13 achieving the predefined ≥75% agreement threshold. This guideline also highlights the need for standardized diagnostic definitions, multicenter registry data, and age-stratified surgical indications not systematically addressed in previously published literature.

## Background

Pediatric flatfoot is one of the most common reasons for orthopedic consultation in childhood (1, 2). Reported prevalence varies widely, from as low as 2.7% to as high as 59%, depending on the age group studied, the diagnostic method used, and the population surveyed (3, 4). More recent studies continue to reflect this variability: Kardm et al. reported a prevalence of 30.06% among school-aged children aged 6–12 years using the Foot Posture Index-6 (43), while Bahir et al. found an overall prevalence of 12.8% among 4,205 schoolchildren aged 7–14 years across urbanized and resource-limited settings (44). In preschool-aged children, flatfoot is nearly universal. The medial longitudinal arch develops progressively during the first decade of life, and most flexible flatfeet resolve spontaneously by age 10 (5, 6). Obesity, ligamentous laxity, male sex, and younger age are consistently identified as associated factors (3, 7, 8). A systematic review and meta-analysis by Xu et al. further confirmed that male sex, age under 9 years, joint laxity, urban environment, and reduced physical activity are significant risk factors (45).

Despite its high prevalence, the management of pediatric flatfoot remains one of the most debated topics in pediatric orthopedics. There is no universally accepted definition. There is no standardized classification system in routine clinical use. Diagnostic criteria differ substantially across studies and across institutions (9, 10). This lack of uniformity extends directly into treatment. Surveys conducted by the European Paediatric Orthopaedic Society and the Italian Pediatric Orthopedics Society have revealed striking variation in clinical practice, even among experienced pediatric orthopedic surgeons (11, 12). The role of foot orthoses, the indications for surgery, the preferred surgical technique, and the optimal age for intervention all remain contested (13, 14).

The evidence base itself contributes to this uncertainty. Cochrane reviews have consistently found insufficient high-quality data to draw firm conclusions about nonsurgical interventions for pediatric flat feet (15, 16). Most surgical studies are retrospective, single-center, and limited to Level III or IV evidence (17, 18). No large multicenter randomized controlled trial has compared conservative and surgical treatment head-to-head with long-term follow-up. The only formal clinical practice guideline, published by the American College of Foot and Ankle Surgeons in 2004, is now over two decades old and predates much of the current literature on subtalar arthroereisis and modern osteotomy techniques (19).

This guideline was therefore developed to address a clear and pressing clinical need. Its aim is to clarify recommendations for the treatment of pediatric flatfoot, with particular attention to the role of operative vs. nonoperative management, tailored to real-world clinical practice. The guideline was produced through a structured process combining a comprehensive systematic review with two rounds of Delphi consensus polling. The expert panel comprised pediatric podiatrists, pediatric orthopedic surgeons, orthotists, and methodology experts. This multidisciplinary composition was chosen to ensure that the final recommendations reflect the full breadth of perspectives involved in the care of children with flatfoot.

The guideline was developed under the Limb Reconstruction Committee of the Orthopedics Branch of China International Exchange and Promotion Association for Medical and Health Care. It is intended for clinicians who evaluate and treat flatfoot in children and teenagers, including orthopedic surgeons, podiatrists, rehabilitation physicians, orthotists, and primary care providers. We hope these recommendations will reduce practice variation, improve clinical decision-making, and identify priority areas for future research.

## Methods

### Study design and setting

A guideline development group (GDG) was established comprising pediatric podiatrists, pediatric orthopedic surgeons, and orthotists (2 pediatric podiatrists, 3 pediatric orthopedic surgeons, and 1 orthotist) from the Limb Reconstruction Committee of Orthopedics Branch of China International Exchange and Promotion Association for Medical and Health Care (CPAM-LRC). The GDG reviewed and finalized the scope of the guideline and agreed on the set of PICOS (population, intervention, comparator, outcomes, study design) for the systematic review. The GDG also completed the list of specialists of the Delphi panel at the first meeting.

### Ethics and registration

This guideline process followed AGREE II principles for transparency and rigor. The systematic review protocol was submitted to PROSPERO for registration prior to manuscript submission (Registration number: CRD420261368215). The systematic review was conducted as per Cochrane Handbook methods. The clinical practice guideline was registered in PREPARE (Practice guideline registration for transparency) (Registration number: PREPARE-2025CN1109) for transparency.

### Systematic review

#### Inclusion and exclusion criteria

The inclusion and exclusion criteria of the included studies are shown in Table 1.

**Table 1:** Inclusion and exclusion criteria.

Items	Inclusion Criteria	Exclusion Criteria
Study Type	Randomized controlled trials (RCTs), non-randomized controlled trials (N-RCTs), quasi-randomized controlled trials, and prospective or retrospective cohort studies involving treatments related to flatfoot.	Reviews, meta-analyses, case reports, case series, expert opinions, editorials, conference abstracts, animal studies, or *in vitro* biomechanical research.
Study Population	Age: Children and adolescents aged ≤18 years. For studies involving mixed populations of adults and children, inclusion is permitted only if data for children can be independently extracted and analyzed. Diagnosis: Patients with a confirmed diagnosis of flexible flatfoot based on clinical examination and/or imaging studies. Symptoms: Participants may be symptomatic or asymptomatic.	Studies involving adults aged >18 years. Diagnosed with rigid flatfoot or secondary flatfoot caused by other identifiable etiologies.
Interventions	The experimental group must include at least one treatment modality, such as: Surgical interventions: Arthrodesis, osteotomy, lateral column lengthening, soft tissue procedures (e.g., tendon transfer/lengthening, Achilles tendon lengthening), arthrodesis, etc. Orthotic devices: Custom or prefabricated foot orthoses/orthotic insoles, heel cups, braces, etc. Immobilization: Casts, splints, etc. Physical therapy: Exercise therapy (e.g., foot muscle stretching, strength training), etc. Others: Functional footwear, night splints, taping therapy, etc. No restrictions on the control group.	
Outcome Measures	The study report must include at least one of the following clinical or radiographic outcome measures: Clinical Measures: Pain scores, functional scores, gait analysis parameters, patient/parent satisfaction, quality of life scores, etc. Radiographic Measures: Arch height, talonavicular angle, calcaneal inclination angle, talonavicular coverage angle, etc.	
Language	English-language literature	
Data and Publication		Duplicate publications (only the most comprehensive or earliest published article will be selected). Studies where full text is unavailable, key data are incomplete, or data extraction is impossible.

#### Literature search strategy

Computerized searches were conducted in PubMed, Embase, Web of Science, and the Cochrane Library databases to collect literature related to flatfoot treatment. The search period spanned from each database's inception to December 10, 2025. Search terms included: flatfoot/flat feet, pes planus, flexible flatfoot, planovalgus; child, pediatric/paediatric, adolescent; orthotic devices, foot orthosis/orthoses, insole/shoe insert, arch support, etc. The specific search strategy is detailed in Supplementary Table 1.

#### Literature screening and data extraction

Two researchers independently performed literature screening, information extraction, and cross-checking. Disagreements were resolved through discussion. EndNote 20 software was used to deduplicate retrieved literature. Based on inclusion and exclusion criteria, titles and abstracts were screened to exclude non-compliant studies. Full-text articles of remaining studies underwent further screening. Data extracted included: study title, first author's name and country, publication date, study type, study population, number of included studies, intervention type, outcome measures, study results, and conclusions.

#### Methodological quality assessment

Two researchers independently assessed the methodological quality of included studies. Disagreements were resolved through discussion, with a third researcher providing final adjudication when necessary. For randomized controlled trials (RCTs), the Cochrane Collaboration's Risk of Bias (RoB) assessment tool was used, evaluating random sequence generation, allocation concealment, blinding, incomplete outcome data, selective reporting, and other biases. Each item was rated as “low risk,” “unclear,” or “high risk.” For non-randomized studies (e.g., cohort studies, comparative studies), the Newcastle–Ottawa Scale (NOS) was used to assess study quality across dimensions including Selection, Comparability, and Outcome/Exposure. Individual study-level risk of bias ratings are presented in Supplementary Table 2.

#### Statistical analysis

Due to the small number of eligible studies and substantial clinical heterogeneity across interventions, populations, and outcome measures, formal meta-analytic pooling of effect estimates was not performed. Instead, results were synthesized narratively and presented using descriptive forest plots to display individual study-level effect sizes (mean differences for continuous outcomes). Flowcharts illustrated the literature screening process. The GRADE framework was applied to assess the certainty of outcome-level evidence across four domains (risk of bias, inconsistency, indirectness, and imprecision). Bubble charts depicted the evidence distribution, presenting information on interventions, outcome measures, effect sizes, and evidence quality. This approach is consistent with Cochrane Handbook guidance for systematic reviews where statistical pooling is inappropriate due to clinical and methodological diversity among included studies.

### Delphi consensus process

In parallel with the evidence review, a multidisciplinary expert panel was convened to formulate recommendations. The panel comprised 20 experts, including pediatric podiatrists, pediatric orthopedic surgeons, orthotists, and methodology experts from CPAM-LRC. We conducted an anonymous electronic Delphi process in two rounds (20). In Round 1, panelists rated proposed statements regarding treatment of pediatric flatfoot on a 4-point Likert scale (Agree, Neutral, Disagree, No Opinion) through a questionnaire by email. After Round 1, statements achieving ≥75% of agreement were considered to have reached consensus; statements not meeting this threshold were revised or clarified based on panel feedback. In Round 2, panelists re-rated the revised statements through an offline consensus meeting. The same ≥75% agreement criterion was applied to define consensus. The process and analysis were guided by established Delphi methods in clinical guideline development. Consensus was predefined as at least 75% of experts rating an item in agreement. All statements meeting the consensus criterion by the end of the second round were accepted as the final recommendations.

It is important to distinguish between two separate assessments used in this guideline. First, certainty of evidence was assessed at the outcome level using the GRADE framework, which evaluates the quality of the body of evidence for each outcome (rated as high, moderate, low, or very low). Individual study-level evidence was classified according to the Oxford Centre for Evidence-Based Medicine (OCEBM) levels (I through V). Second, strength of recommendation was determined independently by the Delphi consensus process, which reflects the degree of expert agreement on each recommendation statement. Specifically, recommendations agreed upon by ≥90% of the expert panel are classified as “High” strength; those agreed upon by 80%–90% as “Moderate”; those agreed upon by 75%–80% as “Mild” (a custom classification adopted for this guideline, analogous to “conditional” in standard guideline terminology); and those with <75% agreement as “No consensus.” This approach ensures that the certainty of the underlying evidence and the degree of expert agreement are reported separately and transparently.

## Results

### Study characteristics

**Figure 1:**
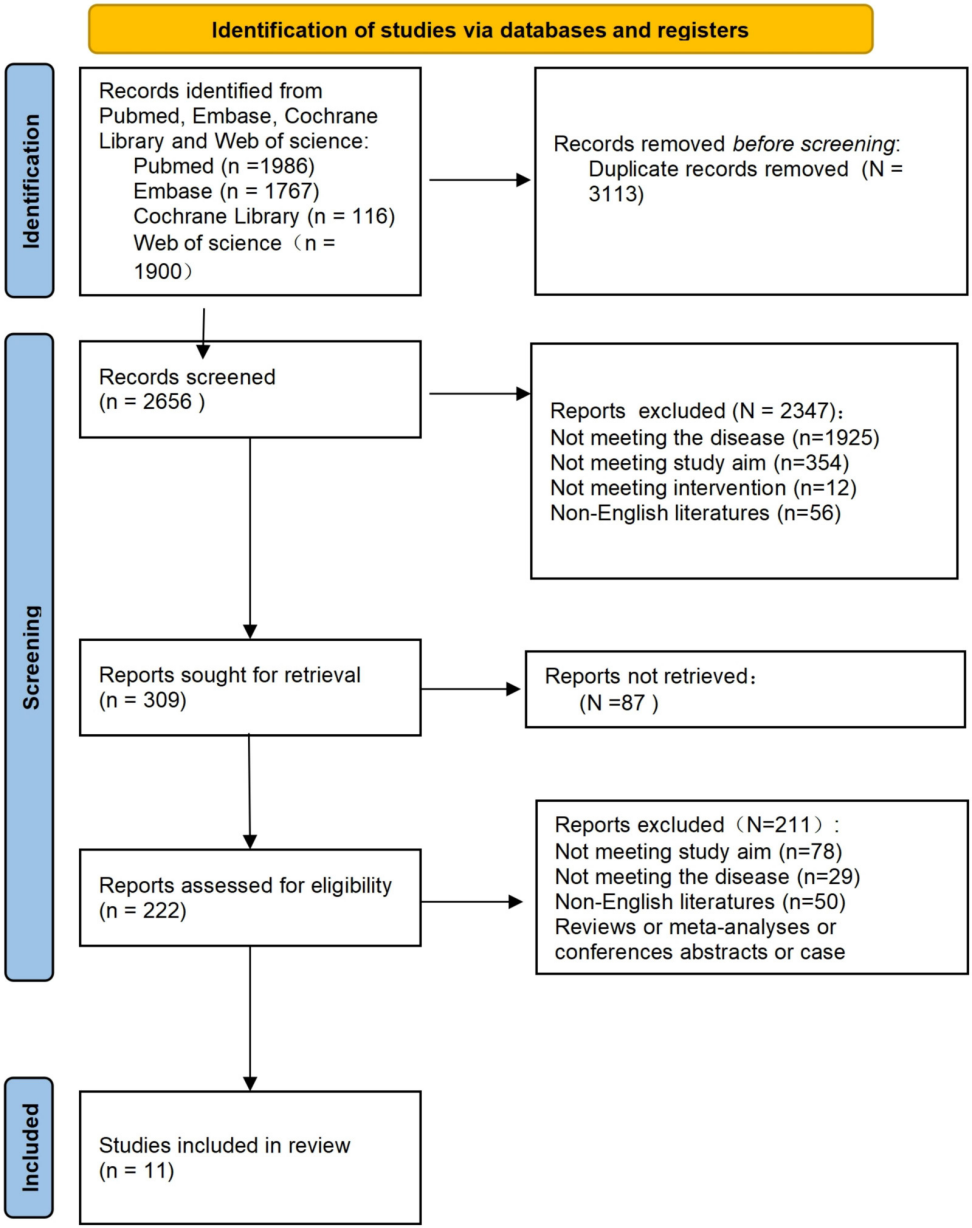
PRISMA flowchart of the studies included. A total of 5,769 records were retrieved from PubMed (*n* = 1,986), Embase (*n* = 1,767), Cochrane Library (*n* = 116), and Web of Science (*n* = 1,900). After removing 3,113 duplicates, 2,656 records underwent title and abstract screening. Initial screening excluded 2,347 records due to non-compliance with disease criteria (*n* = 1,925), research objectives (*n* = 354), intervention measures (*n* = 12), or non-English language (*n* = 56). A total of 309 full texts were requested, of which 87 were unavailable. Ultimately, 222 full texts underwent eligibility assessment, resulting in the exclusion of 211 studies (*n* = 78 for non-alignment with research objectives, *n* = 29 for non-alignment with disease criteria, *n* = 50 for non-English language, and others including reviews, meta-analyses, conference abstracts, and case reports). A total of 11 studies were finally included.

A total of 5,769 records were retrieved from PubMed (*n* = 1,986), Embase (*n* = 1,767), Cochrane Library (*n* = 116), and Web of Science (*n* = 1,900). After removing 3,113 duplicates, 2,656 records underwent title and abstract screening. Initial screening excluded 2,347 records due to non-compliance with disease criteria (*n* = 1,925), research objectives (*n* = 354), intervention measures (*n* = 12), or non-English language (*n* = 56). A total of 309 full texts were requested, of which 87 were unavailable. Ultimately, 222 full texts underwent eligibility assessment, resulting in the exclusion of 211 studies (*n* = 78 for non-alignment with research objectives, *n* = 29 for non-alignment with disease criteria, *n* = 50 for non-English language, and others including reviews, meta-analyses, conference abstracts, and case reports). The process and results of literature screening are shown in Figure 1.

This systematic review included a total of 11 primary studies (21–31), comprising 5 randomized controlled trials (21, 26–28, 30) and 6 observational studies [3 retrospective comparative studies (22, 23, 31), 2 cohort studies (24, 29), and 1 single-center cohort study (25)]. Sample sizes ranged from 30 to 402 participants; the research timeframe spanned approximately 1995 to 2019, with some studies failing to report their specific study periods. Interventions included orthotic/insoles-related interventions, rehabilitation therapies, and surgical techniques. Primary outcome measures encompassed foot and ankle function and pain scores, imaging/radiographic measurements, and gait biomechanical parameters. The basic characteristics of all 11 included studies are shown in Table 2. In addition to the 11 formally included primary studies, the guideline recommendations draw upon published systematic reviews, meta-analyses, and expert society surveys (11, 12, 14–18, 32–42) that did not meet the primary study inclusion criteria but provided essential contextual evidence for formulating clinical recommendations.

**Table 2:** Basic characteristics of included studies.

Study	Country/Region	Study design	Study period	Population (*n*)	Intervention/Control	Primary Outcome
Liebau et al. (21)	Germany	Randomized Controlled Trial	NR	52	Supportive: sensory insole vs. placebo insole	FADI; valgus index; static/dynamic plantar contact area; MVC (or MVC-related muscle activity)
Memeo et al. (22)	Italy	Comparative Study	2003–2011	402	Surgery vs. surgery	Pain scale; CB (Costa–Bertani angle); HI (heel inclination angle); TDA (talar declination angle); KI (Kite angle).
Moraleda et al. (23)	Spain:USA	Retrospective Comparative Study	1996–2009	63	Surgery vs. surgery	AP talo–first MTT angle; AP talocalcaneal angle; talonavicular coverage; lateral (L) talo–first MTT angle; calcaneal pitch; lateral (L) talocalcaneal angle
Hu et al. (24)	China	Retrospective Cohort Study	2013–2019	31	Surgery vs. surgery vs. surgery	AP talo–first MTT angle; AP talocalcaneal angle; talonavicular coverage; lateral talo–first MTT angle; calcaneal pitch; lateral talocalcaneal angle
Vogt et al. (25)	Germany	Single-center Cohort Study	2002– 2013	73	Surgery vs. surgery vs. surgery	Treatment satisfaction
Tahririan et al. (26)	Iran	Randomized Controlled Trial	2018–2019	66	Surgery vs. surgery	AP talus–1st metatarsal angle; lateral talus–1st metatarsal angle; calcaneal pitch; AOFAS score; VAS pain score
Ahn et al. (27)	Korea	Randomized Controlled Trial	NR	40	Orthosis vs. orthosis	Anteroposterior talocalcaneal angle (APTCA); Lateral talocalcaneal angle (LTTCA); Lateral talometatarsal angle (LTTMA); Calcaneal Pitch (CP); Resting calcaneal stance position (RCSP)
Jafarnezhadgero et al. (28)	Iran	Randomized Controlled Trial	2017–2018	30	Orthosis vs. insole	kinematics (ankle: knee: hip angles); kinetics (GRF)
Riccio et al. (29)	Italy	Prospective Cohort Study	1995–1997	300 (600 feet)	Rehabilitation therapy vs. orthopedic insole	Improvement in foot arch shape
Hsieh et al. (30)	Taiwan	Randomized Controlled Trial	2015–2015	52	Insole vs. no treatment	PODCI (pain/comfort; transfer & basic mobility; sports & physical function; global function; happiness); PedsQL (physical health summary; psychosocial health summary; total score); Timed Up and Go; 10-m fast walking time; stair ascent/descent time; chair rise time
Sever et al. (31)	Türkiye	Retrospective Comparative Study	2015–2017	50	Foot orthosis + taping vs. foot orthosis	AOFAS; radiographic measures; adverse events

NR, Not report; FADI, Foot and Ankle Disability Index; MVC, Maximum Voluntary Contraction; CB, Costa–Bertani angle; HI, Heel Inclination angle; TDA, Talar Declination Angle; KI, Kite angle; AP, Anteroposterior; MTT, Metatarsal; L, Lateral; AOFAS, American Orthopaedic Foot and Ankle Society; VAS, Visual Analog Scale; APTCA, Anteroposterior Talocalcaneal Angle; LTTCA, Lateral Talocalcaneal Angle; LTTMA, Lateral Talometatarsal Angle; CP, Calcaneal Pitch; RCSP, Resting Calcaneal Stance Position; GRF, Ground Reaction Force; PODCI, Pediatric Outcomes Data Collection Instrument; PedsQL, Pediatric Quality of Life Inventory.

### Outcomes

#### Conservative interventions

Among the included studies evaluating conservative interventions, Liebau et al. conducted a double-blind randomized controlled trial comparing supportive insoles with sensory insoles and placebo insoles in 52 children with flexible flatfoot (21). No significant between-group differences were observed in the Foot and Ankle Disability Index, valgus index, or static and dynamic plantar contact area, suggesting that the type of insole support did not differentially influence foot structure or function over the study period. Hsieh et al. conducted a randomized controlled trial of 52 symptomatic children and demonstrated that customized arch support insoles produced significant improvements in pain and comfort scores and physical health over 12 weeks compared to no treatment (30). Ahn et al. compared rigid foot orthoses with talus control foot orthoses over 12 months in 40 children and found that talus control orthoses were significantly superior for correction of the anteroposterior talocalcaneal angle and resting calcaneal stance position (27). Jafarnezhadgero et al. showed in a randomized controlled trial of 30 boys that 4 months of medial arch support orthosis use produced significant improvements in ankle eversion, knee abduction, and ground reaction forces during walking (28).

Regarding rehabilitation, Riccio et al. conducted a prospective cohort study of 300 children (600 feet) with bilateral flexible flatfoot treated with structured rehabilitative exercises over a mean period of 2.75 years (29). Normalization rates of 91.2% for Viladot type III feet (moderate deformity) and 98.1% for Viladot type II feet (mild deformity) were reported, compared to 54.0% and 89.2%, respectively, in a historical cohort treated with orthoses alone. Sever et al. compared UCBL orthoses with and without Kinesio Taping in 50 juvenile athletes and found that the combination achieved a lower complication rate (10% vs. 52%) with improved patient compliance (31).

#### Surgical interventions

**Figure 2:**
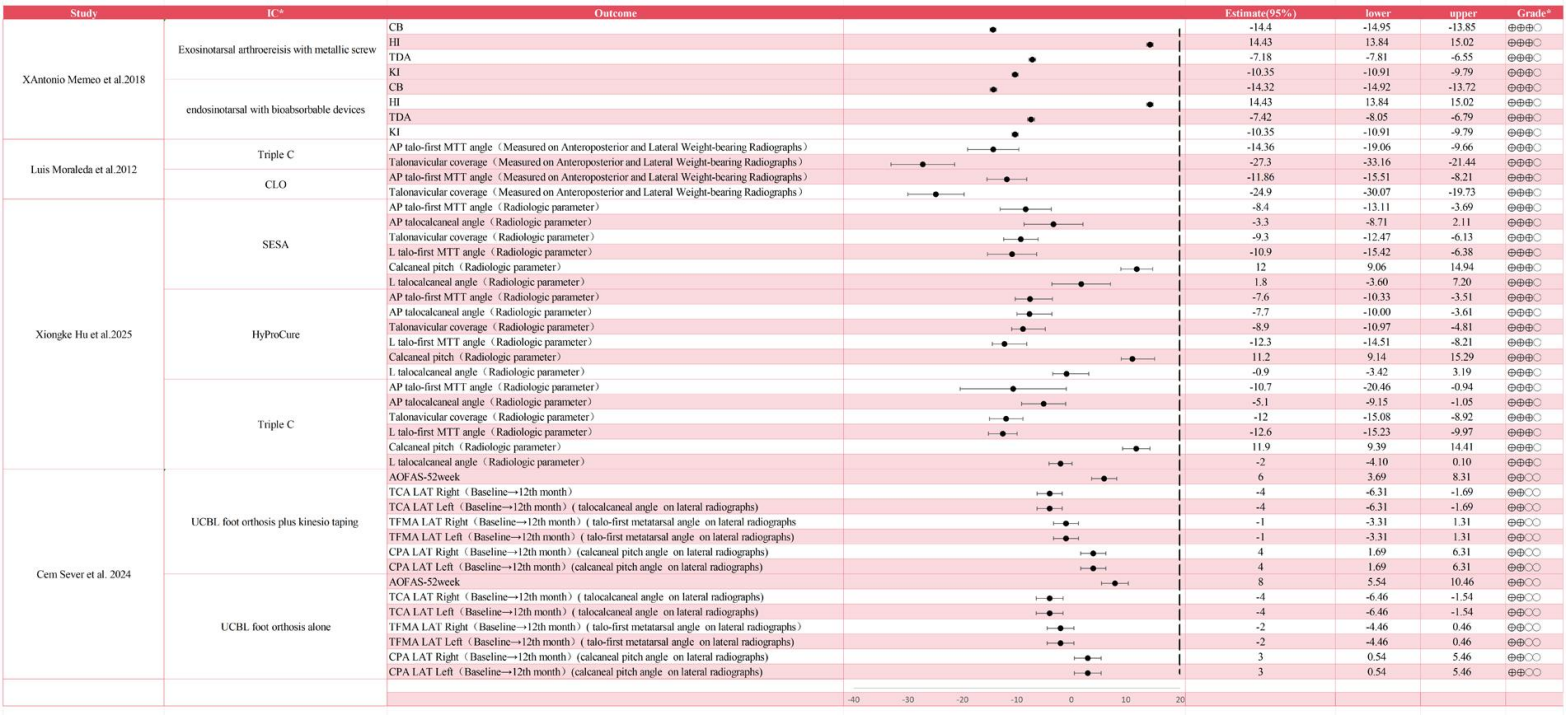
Descriptive forest plot of related studies. IC*:intervention or control; Grade*: Very low qualiy: ⊕OOO; Low qualiy: ⊕⊕OO; Moderate qualiy: ⊕⊕⊕ O; High qualiy: ⊕⊕⊕⊕; Triple C, Calcaneo-Cuboid-Cuneiform Osteotomies; CLO, the calcaneal-lengthening osteotomy; SESA, Subtalar extra-articular screw arthroereisis; HyProCure, HyProCure implantation at tarsal sinus; CB, Costa Bertani angle; HI, heel inclination angle; TDA, talar declination angle; KI, kite angle; AP, anteroposterior; MTT, metatarsal; L, lateral; ACFAS, American College of Foot and Ankle Surgeons score; TCA LAT, talocalcaneal angle on lateral radiographs; TFMA LAT, talo-first metatarsal angle on lateral radiographs; CPA LAT, calcaneal pitch angle on lateral radiographs.

The data synthesis for surgical outcomes involves four observational studies, which assess interventions spanning from minimally invasive subtalar arthroereisis and corrective osteotomies to lateral column lengthening. This analysis centers on quantifying the magnitude of the effect on radiographic parameters, including the Costa Bertani angle, talar declination angle, and talonavicular coverage, as well as clinical functional scores. The aim is to evaluate the extent of anatomical restoration and functional improvement offered by each intervention, thereby establishing a quantitative baseline for the comprehensive efficacy assessment. Figure 2 shows the analysis of continuous variables obtained from non-randomized controlled trials incorporated in this review.

In the realm of subtalar arthroereisis, Antonio Memeo et al. conducted a comparison between metallic screws and bioabsorbable devices for exosinotarsal and endosinotarsal arthroereisis (22). The outcomes revealed a high level of consistency between the two materials in terms of continuous radiographic results. Specifically, both groups exhibited a reduction in the Costa Bertani angle of approximately 14 degrees, an increase in the heel inclination angle of roughly 14.4 degrees, and a decrease in the talar declination angle of about 7 degrees. These findings imply that the corrective mechanism predominantly depends on the mechanical obstruction of the sinus tarsi rather than the material characteristics of the implant. Xiongke Hu et al. further assessed Subtalar Extra-articular Screw Arthroereisis (SESA) against HyProCure implantation (24). Both interventions led to significant enhancements in calcaneal pitch, measuring 12 degrees and 11.2 degrees respectively. Nevertheless, the confidence interval for the lateral talocalcaneal angle in the SESA group crossed zero (1.8, 95% CI: −3.60 to 7.20), suggesting that SESA may have less statistical stability in correcting hindfoot valgus compared to the HyProCure system.

Research conducted by Luis Moraleda et al. and Xiongke Hu et al. both delved into the Calcaneo-Cuboid-Cuneiform Osteotomies (Triple C) (23, 24). As a reliable reconstructive alternative, Moraleda's findings suggested that the Triple C procedure attained a considerable effect size in rectifying talonavicular coverage (−27.3), surpassing the calcaneal lengthening osteotomy (−24.9) and significantly outperforming the effect noted in Hu's study cohort for the same procedure (−12). This disparity presumably reflects the differences in the severity of baseline deformities among the study populations. Overall, osteotomies exhibited the most effective anatomical reconstruction for correcting forefoot abduction, as manifested by continuous metrics such as talonavicular coverage and the AP talo-first metatarsal angle, with radiographic alterations generally exceeding those achieved by isolated arthroereisis.

### The risk of bias and study quality

**Figure 3:**
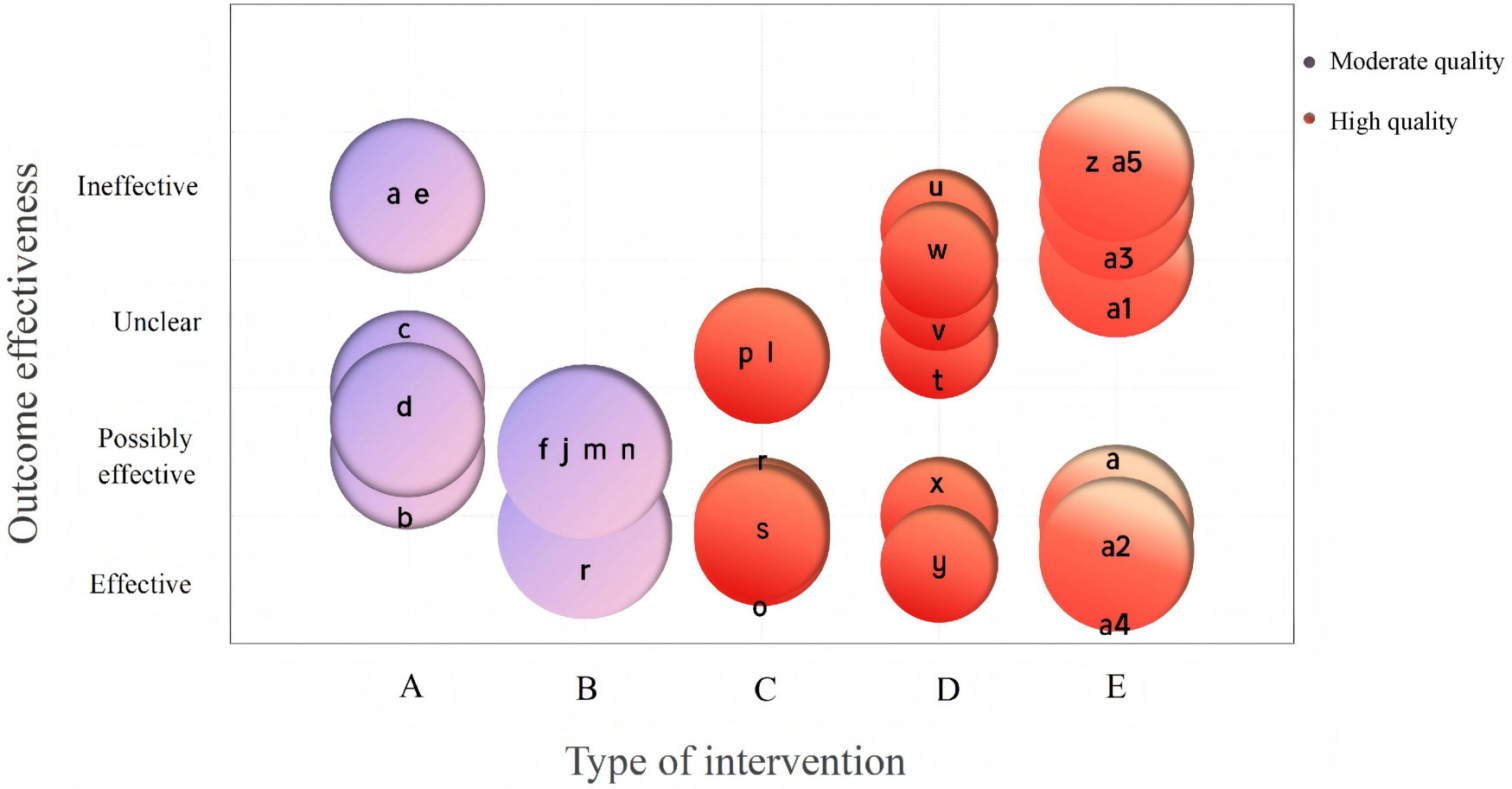
Evidence Map of treatment strategies for flat feet. Intervention measures: A: Supportive/Comfortable Insoles; B: Operative treatment; C: Talus control foot orthosis (TCFO); D: Arch support foot orthoses; E: Arch-supporting insole. Outcome indicator: a: Pain; b: Function; c: Foot alignment (Valgus index); d: Myoelectricity; e: Contact area; f: AP Talus-1st Metatarsal Angle; j: Lat Tal-1st Metatarsal Angle; m: Ankle Society; n: VAS Pain; o: Anteroposterior talocalcaneal angle (APTCA); p: Lateral talocalcaneal angle (LTTCA); l: Lateral talometatarsal angle (LTTMA); *r*: Calcaneal Pitch (CP); s: Resting calcaneal stance position (RCSP); t: Maximum ankle plantarflexion angle during loading response; u: Maximum ankle dorsiflexion angle during mid-stance; v: Maximum ankle plantarflexion angle at toe-off; w: Maximum ankle inversion angle; x: Maximum frontal angle of the ankle during push-off; y: Maximum ankle internal rotation angle; z: Normal speed walking; a1: Chair rising; a2: Upper extremity and physical function; a3: Happiness; a4: Physical; a5: Psychosocial.

The bubble chart design presents included studies across five dimensions (number of participants, flatfoot intervention type, outcome measures, outcome effectiveness, and quality assessment results), enabling intuitive and effective observation of the efficacy of different flatfoot treatment strategies (Figure 3). Each bubble represents an outcome measure, with different colors indicating varying levels of evidence quality for that outcome. Bubble size reflects the number of participants in randomized controlled trials (RCTs). The horizontal axis displays different intervention types for flatfoot treatment; the vertical axis displays the effectiveness of the corresponding outcome measure for each intervention type, categorized based on study conclusions as: clear benefit, probable benefit, unclear benefit, or no benefit. GRADE assessment of outcome evidence quality indicates mostly low to moderate quality, with only one high-quality outcome. Individual study-level risk of bias ratings are provided in Supplementary Table 2.

### Delphi round

Eighteen preliminary consensus statements were drafted based on the systematic review by the GDG. At the first Delphi round, all 18 preliminary consensus statements were sent to the Expert Panel via email. All of the participants (20/20) responded to the 18 consensus statements. Ten statements (55.6%) met the 75% criteria of agreement for consensus. Six statements (33.3%) were revised by the GDG based on the suggestions by the Expert Panel. Two statements (11.1%) were removed, leaving 16 statements for the second round.

**Figure 4:**
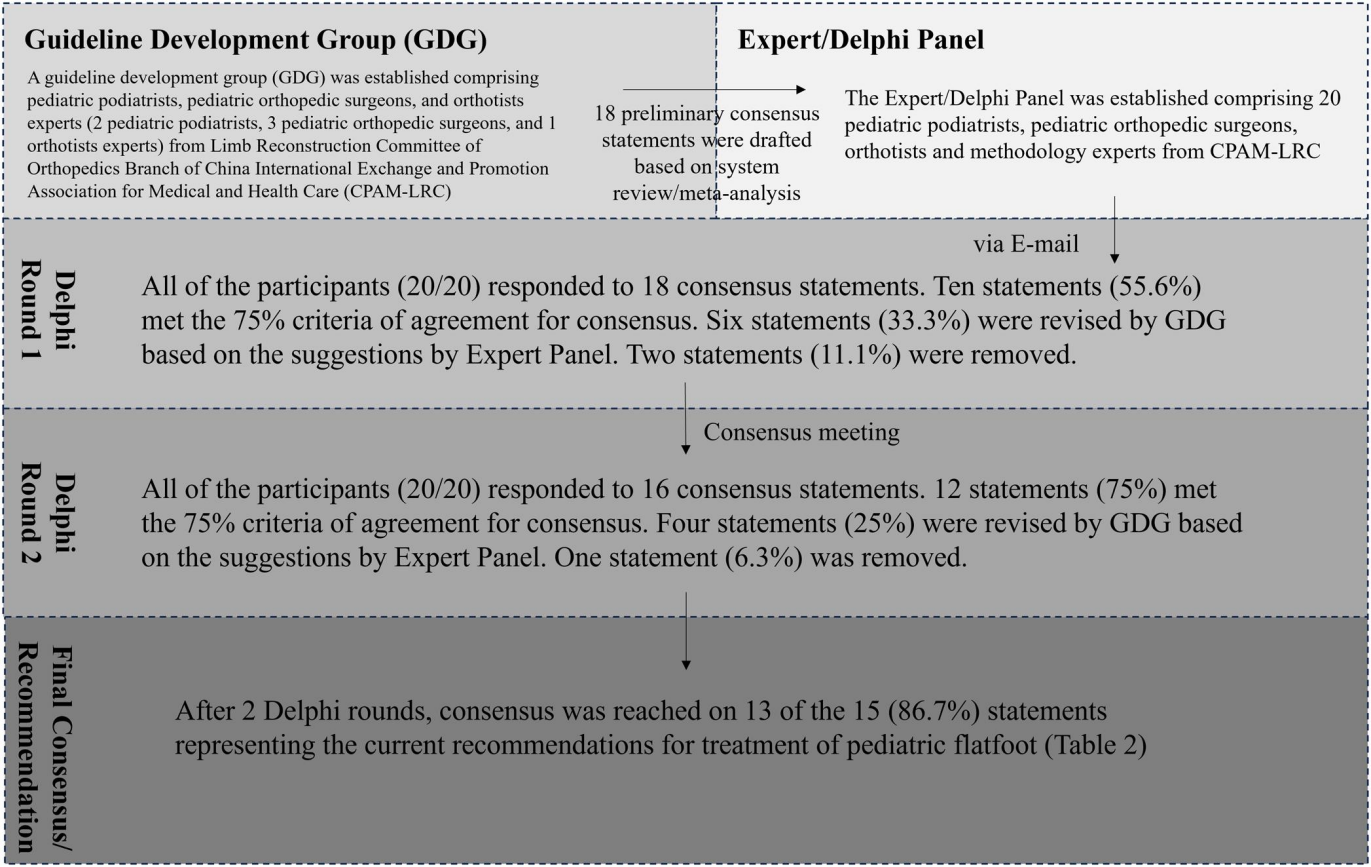
Flow diagram of the Delphi round. Eighteen preliminary consensus statements were drafted based on systematic review by GDG. At first Delphi round, all 18 preliminary consensus statements were sent to Expert Panel via Email. All of the participants (20/20) responded to 18 consensus statements. Ten statements (55.6%) met the 75% criteria of agreement for consensus. Six statements (33.3%) were revised by GDG based on the suggestions by Expert Panel. Two statements (11.1%) were removed. At second Delphi round, an offline consensus meeting was held and all 20 participants of Expert Panel were presented. Twelve statements (75%) met the 75% criteria of agreement for consensus. Four statements (25%) were revised by GDG based on the suggestions by Expert Panel. One statement (6.3%) was removed. After 2 Delphi rounds, consensus was reached on 13 of the 15 statements (86.7%) representing the current recommendations for treatment of pediatric flatfoot.

At the second Delphi round, an offline consensus meeting was held and all 20 participants of the Expert Panel were present. Of the 16 statements presented, 12 (75.0%) met the 75% criteria of agreement for consensus. Of the 4 that did not reach consensus, 3 were revised by the GDG based on the Expert Panel's discussion and retained in the final recommendation set, while 1 was removed. After 2 Delphi rounds, 15 recommendation statements were finalized. Of these, 13 (86.7%) achieved the predefined ≥75% agreement threshold (the 12 that reached consensus at Round 2, plus 1 of the 3 revised statements that achieved consensus upon re-evaluation). Two statements (Recommendations 10 and 12) did not reach consensus but are retained for completeness with a clear label indicating that expert consensus was not achieved (Figure 4). The Delphi consensus statements and level of agreement at the final meeting are shown in Table 3.

**Table 3:** Delphi consensus statement and level of agreement at final meeting.

Recommendation of treatment of pediatric flatfoot	Agree	Neutral	Disagree	No opinion
Domain A: natural history and timing of intervention				
**Recommendation 1. Observation for asymptomatic flexible flatfoot.** Asymptomatic flexible flatfoot in children under age 6 is a physiological variant that resolves spontaneously in the majority of cases and does not require treatment. Parental reassurance and clinical observation are the recommended approach. Routine use of orthoses or corrective footwear in asymptomatic children is not supported by evidence (1, 2, 6, 15).	100%	0	0	0
**Recommendation 2. Clinical reassessment at age 8 to 10 years.** Children with persistent flexible flatfoot should undergo structured clinical reassessment around age 8 to 10, as most spontaneous arch development occurs during the first decade. After age 10, minimal further spontaneous improvement is expected, and persistent symptomatic flatfoot may warrant active intervention (2, 6).	75%	10%	5%	10%
**Recommendation 3. Indications for initiating treatment**. Active treatment, whether conservative or surgical, should be considered when flexible flatfoot is accompanied by one or more of the following: pain at the plantar arch, hindfoot, or midfoot; functional limitation or fatigue during walking and sport; rapid progression of deformity; or associated equinus contracture. Pain, functional impairment, and age are the three most important clinical parameters guiding treatment decisions (2, 6, 11, 12).	90%	5%	0	5%
Domain B: conservative treatment				
**Recommendation 4. Rehabilitative exercises as first-line conservative therapy.** Structured rehabilitative exercise programs, including stretching of the gastrocnemius-soleus complex and peroneal muscles, proprioceptive training, and arch-strengthening exercises, should be offered as first-line treatment for symptomatic flexible flatfoot. Exercise programs have been shown to be more effective than orthoses alone, with normalization rates of 91.2% for Viladot type III feet (moderate deformity, defined by plantar contact area between one-half and two-thirds of total footprint width) and 98.1% for Viladot type II feet (mild deformity, defined by plantar contact area between one-third and one-half of total footprint width) (2, 29).	75%	15%	5%	5%
**Recommendation 5. Foot orthoses for symptomatic flatfoot only.** Foot orthoses should be prescribed for symptomatic relief in children with symptomatic flexible flatfoot only, and not for the purpose of structural arch correction. There is no evidence that orthoses alter the structural development of the medial longitudinal arch. Prefabricated orthoses are recommended as the default for routine flexible flatfoot. Custom orthoses should be reserved for atypical foot morphology, specific conditions such as juvenile idiopathic arthritis, or failure to respond to prefabricated devices (15, 18, 21, 30, 32–34).	90%	0	0	10%
**Recommendation 6. Type of foot orthosis and adjunct therapies.** When orthoses are indicated, talus control foot orthoses with talonavicular joint coverage are recommended as the preferred type, given their superior correction of hindfoot alignment compared to standard rigid foot orthoses. UCBL orthoses combined with Kinesio Taping may improve compliance and reduce pressure-related complications, particularly in athletic children. Long-term use of arch support orthoses, sustained for at least 3 to 4 months, improves lower-limb biomechanics during walking (27, 28, 31).	80%	10%	5%	5%
**Recommendation 7. Duration of conservative treatment before considering surgery.** A minimum of 6 months of structured conservative treatment, encompassing orthoses, rehabilitative exercises, activity modification, and weight management if applicable, should be attempted before considering surgical intervention. Only patients who remain symptomatic after adequate conservative management should be referred for surgical evaluation (11, 14, 17).	90%	0	0	10%
Domain C: surgical treatment — indications and timing				
**Recommendation 8. Indications for surgical treatment.** Surgery is indicated for symptomatic flexible flatfoot that has failed at least 6 months of adequate conservative treatment, with persistent pain or functional impairment. Fewer than 10% of children with flexible flatfoot are surgical candidates. Additional indications include progressive deformity, associated equinus contracture unresponsive to stretching, and severe deformity with hindfoot valgus greater than 15 degrees or complete arch collapse. Rigid flatfoot and tarsal coalition must be excluded prior to surgical planning (2, 6, 11, 17, 38).	75%	10%	0	15%
**Recommendation 9. Age considerations for surgery.** Subtalar arthroereisis is typically considered between ages 8 and 14 years to allow sufficient skeletal maturation while preserving the remodeling potential of growth. Bony osteotomy procedures, including calcaneal lengthening, medializing calcaneal osteotomy, triple C, and Cotton osteotomy, are generally reserved for children aged 10 years and older, or for adolescents with severe deformity. Joint-sparing procedures are preferred over arthrodesis in the pediatric population to preserve joint mobility (17, 22–24, 36, 40).	75%	10%	5%	10%
Domain D: surgical procedures				
**Recommendation 10. Subtalar arthroereisis as a minimally invasive option.** [**No consensus**]**.** Subtalar arthroereisis, including the calcaneo-stop technique, is a valid minimally invasive surgical option for moderate symptomatic flexible flatfoot in children. It produces excellent patient satisfaction in approximately 80% of cases, significant improvement in all radiographic parameters, and a complication rate of approximately 7%. The choice of arthroereisis implant type does not significantly affect long-term outcomes; however, implant-specific complication profiles differ and should be discussed with families. Note: Expert consensus was not reached (≥75% threshold not met); this does not indicate that the procedure is clinically contraindicated (17, 22, 24, 25, 35, 36).	55%	20%	15%	10%
**Recommendation 11. Lateral column lengthening for moderate-to-severe deformity.** Lateral calcaneal lengthening osteotomy achieves greater radiographic correction than arthroereisis, particularly for forefoot abduction and talonavicular coverage. It is appropriate for moderate-to-severe deformity, especially when significant forefoot abduction is present. Surgeons should be aware that calcaneocuboid subluxation is a frequent radiographic finding, occurring in up to 50% of cases following this procedure, though clinical significance varies (23, 26, 37).	75%	10%	5%	10%
**Recommendation 12. Combined and tailored surgical approaches for severe deformity.** [**No consensus**]**.** Severe flexible flatfoot, defined by hindfoot valgus greater than 15 degrees with significant forefoot abduction and residual forefoot supination, may require a combination of procedures tailored to the individual deformity pattern. These include medializing calcaneal osteotomy for hindfoot valgus correction, lateral column lengthening for forefoot abduction, Cotton osteotomy for residual forefoot supination, and soft tissue procedures as needed. This multiplanar approach achieves full correction while avoiding arthrodesis in the growing skeleton. Note: Expert consensus was not reached (≥75% threshold not met); this does not indicate that the approach is clinically contraindicated (39–41).	65%	20%	10%	5%
**Recommendation 13. Role of soft tissue procedures as adjuncts.** Gastrocnemius recession or Achilles tendon lengthening should be performed when equinus contracture is present, defined as ankle dorsiflexion less than 10 degrees with the knee extended. For severe deformities, medial soft tissue tightening, including spring ligament repair and talonavicular capsule plication, may be required in addition to arthroereisis or osteotomy to adequately correct forefoot abduction and maintain correction. Additional soft tissue procedures are not recommended for mild-to-moderate deformities already corrected by osteotomy alone (6, 23, 39, 41).	75%	15%	5%	5%
Domain E: outcomes and follow-up				
**Recommendation 14. Outcome assessment and standardized measures.** Treatment outcomes should be evaluated using a combination of validated patient-reported outcome measures such as the AOFAS score, Foot Function Index, Visual Analogue Scale for pain, and PODCI for pediatric function, together with radiographic parameters including Meary's angle, calcaneal pitch, talonavicular coverage angle, and anteroposterior talocalcaneal angle. Pedobarography is recommended as a radiation-free adjunct for monitoring treatment response where available. Follow-up should include assessment at a minimum of 6 months, 1 year, and 2 years post-intervention, with longer follow-up of at least 4 years recommended for surgical patients to capture long-term outcomes and detect recurrence (11, 25, 26, 38).	90%	0	0	10%
**Recommendation 15. Need for high-quality evidence and registry data.** Current evidence for pediatric flatfoot treatment is predominantly Level III to IV, with considerable heterogeneity in diagnostic criteria, interventions, and outcome measures across studies. No large multicenter randomized controlled trials compare conservative versus surgical treatment or different surgical techniques head-to-head with long-term follow-up. Future research should prioritize multicenter prospective trials with standardized diagnostic criteria, validated outcome measures, and minimum 5-year follow-up. Establishment of multicenter registries is recommended to accumulate sufficient data for evidence-based guideline refinement (11, 15, 17, 38).	90%	0	0	10%

Agree: 75%–100% consensus agreement; Neutral: 50%–74% consensus agreement; Disagree: 0%–49% consensus agreement.

“No consensus” indicates that expert agreement did not reach the predefined ≥75% threshold; it does not indicate that the procedure or approach is clinically contraindicated.

For each Delphi round, specialists from the Delphi panel were suggested to give recommendation based on the results of our systematic review and their own clinical experience. The recommendation of “Agree” usually refers to both our systematic review and their own clinical experience are consistent with the recommendation. The recommendation of “Neutral” usually refers the specialist has limited experience and noted some contraindication of the result of systematic review. “Disagree” usually refers the specialist has great clinical experience and noted obvious contraindication of the result of systematic review. No opinion refers the specialist has no clinical experience (usually regarding some certain technique).

### Statements of guidelines with level of recommendation

#### Recommendation 1. Observation for asymptomatic flexible flatfoot

We recommend observation and parental reassurance as the standard approach for asymptomatic flexible flatfoot in children under age 6, and continued observation throughout the first decade for children who remain asymptomatic. The medial longitudinal arch develops spontaneously during the first decade of life, and most children achieve a normal arch by age 10 without any intervention (5, 6). A Cochrane review of 16 randomized controlled trials involving 1,058 children found no evidence that foot orthoses alter the natural arch development in painless flexible flatfoot (15). Routine use of orthoses or corrective footwear in asymptomatic children is not supported by evidence (1, 2) (Table 3).

*Level of recommendation: High*

#### Recommendation 2. Clinical reassessment at age 8 to 10 years

We recommend structured clinical reassessment for children with persistent flexible flatfoot at age 8 to 10 years, as most spontaneous arch development occurs during the first decade and minimal further improvement is expected after age 10. Longitudinal studies have confirmed that the arch typically stabilizes by this age (5, 6). A cross-sectional study of 835 school-aged children demonstrated that flatfoot prevalence decreased from 54% at age 3 to approximately 26% at age 6, with limited further change beyond age 10 (3). Mosca, in a narrative review, emphasized that persistent deformity at this stage should prompt structured reassessment for symptoms, functional impairment, and associated equinus contracture (6). This age window represents a critical decision point for determining whether active intervention is warranted (2).

*Level of recommendation: Mild*

#### Recommendation 3. Indications for initiating treatment

We recommend initiating active treatment, whether conservative or surgical, only when flexible flatfoot is accompanied by one or more of the following: pain at the plantar arch, hindfoot, or midfoot; functional limitation or fatigue during walking and sport; rapid progression of deformity; or associated equinus contracture. The European Paediatric Orthopaedic Society survey of 140 specialists identified pain, age, and ligamentous laxity as the three most important clinical parameters guiding treatment decisions (11). The Italian Pediatric Orthopedics Society survey confirmed that plantar arch pain was considered the most critical factor by 61.8% of respondents, followed by fatigue during walking at 59.1% (12). Mosca, in a narrative review, further identified associated equinus contracture as the primary pathological subtype linked to symptoms and functional limitation (6). Rapid progression of deformity constitutes an additional indication (2).

*Level of recommendation: High*

#### Recommendation 4. Rehabilitative exercises as first-line conservative therapy

We recommend structured rehabilitative exercise programs, including gastrocnemius-soleus stretching, proprioceptive training, and intrinsic foot muscle strengthening, as first-line conservative treatment for symptomatic flexible flatfoot, particularly for moderate deformities. Riccio and colleagues conducted a prospective cohort study of 300 children (600 feet) with bilateral flexible flatfoot treated with structured rehabilitative exercises over a mean period of 2.75 years (29). At follow-up, 91.2% of Viladot type III feet (moderate deformity, characterized by a plantar surface contact area between one-half and two-thirds of the total footprint width) normalized with rehabilitation, compared to only 54.0% in a historical cohort treated with orthoses alone. For Viladot type II feet (mild deformity, characterized by a plantar contact area between one-third and one-half of the total footprint width), the normalization rate was 98.1% with exercises vs. 89.2% with orthoses (29).

*Level of recommendation: Mild*

#### Recommendation 5. Foot orthoses for symptomatic flatfoot only

We recommend that foot orthoses be prescribed only for symptomatic relief in children with symptomatic flexible flatfoot and not for the purpose of structural arch correction. Prefabricated orthoses are recommended as the default option for routine flexible flatfoot; custom orthoses should be reserved for atypical foot morphology, specific conditions such as juvenile idiopathic arthritis, or failure to respond to prefabricated devices. The 2022 Cochrane review analyzed 16 randomized controlled trials and concluded that custom foot orthoses provided no significant benefit over shoes alone for painless flat feet in otherwise healthy children (15). An updated systematic review by Dars and colleagues similarly reported that evidence for structural improvement with orthoses remained weak and inconsistent (18). A systematic review and meta-analysis of patient-reported outcomes found no significant benefit of foot orthoses in children with flexible flatfoot (33). However, Hsieh and colleagues demonstrated that customized arch support insoles produced significant improvements in pain and comfort scores and physical health over 12 weeks in symptomatic children (30). The current evidence supports orthosis prescription for symptomatic relief only (15, 21, 32, 34).

*Level of recommendation: High*

#### Recommendation 6. Type of foot orthosis and adjunct therapies

When orthoses are indicated, we recommend talus control foot orthoses with talonavicular joint coverage as the preferred type, given their superior correction of hindfoot alignment compared to standard rigid foot orthoses. UCBL orthoses combined with Kinesio Taping may be considered as an adjunct to improve compliance and reduce pressure-related complications, particularly in athletic children. Long-term use of arch support orthoses, sustained for at least 3 to 4 months, improves lower-limb biomechanics during walking. Ahn and colleagues conducted a randomized controlled trial of 40 children comparing rigid foot orthoses with talus control foot orthoses over 12 months (27). Talus control orthoses were significantly superior for correction of the anteroposterior talocalcaneal angle and resting calcaneal stance position. Jafarnezhadgero and colleagues showed in a randomized controlled trial of 30 boys that 4 months of medial arch support orthosis use produced significant improvements in ankle eversion, knee abduction, and ground reaction forces during walking (28). Sever and colleagues reported that UCBL orthoses combined with Kinesio Taping achieved a lower complication rate of 10% compared to 52% with UCBL alone, along with improved patient compliance (31).

*Level of recommendation: Moderate*

#### Recommendation 7. Duration of conservative treatment before considering surgery

We recommend a minimum of 6 months of structured conservative treatment, encompassing orthoses, rehabilitative exercises, activity modification, and weight management if applicable, before considering surgical intervention. Only patients who remain symptomatic after adequate conservative management should be referred for surgical evaluation. The European Paediatric Orthopaedic Society survey found that the vast majority of specialists endorsed a minimum trial of conservative treatment before considering surgery (11). Bernasconi and colleagues in their comprehensive review recommended at least 6 months of structured conservative management before surgical referral (14). Smith and colleagues confirmed this threshold in their systematic review of subtalar arthroereisis outcomes, noting that all included studies required failure of at least 6 months of conservative treatment as a surgical inclusion criterion (17).

*Level of recommendation: High*

#### Recommendation 8. Indications for surgical treatment

We recommend surgical treatment for symptomatic flexible flatfoot that has failed at least 6 months of adequate conservative treatment, with persistent pain or functional impairment. Fewer than 10% of children with flexible flatfoot are surgical candidates. Additional indications include progressive deformity, associated equinus contracture unresponsive to stretching, and severe deformity with hindfoot valgus greater than 15 degrees or complete arch collapse. Rigid flatfoot and tarsal coalition must be excluded through clinical examination and imaging before surgical planning, as these conditions require fundamentally different management strategies. The EPOS survey reported that 82% of respondents felt fewer than 10% of children with flexible flatfoot are surgical candidates (11). Persistent pain, functional impairment, and failure of at least 6 months of conservative treatment constitute the primary indications (6, 17). A systematic review of long-term surgical outcomes identified severe hindfoot valgus exceeding 15 degrees and complete arch collapse as additional indicators for surgical intervention (38).

*Level of recommendation: Mild*

#### Recommendation 9. Age considerations for surgery

We recommend that subtalar arthroereisis be considered between ages 8 and 14 years to balance skeletal maturity with growth remodeling potential, while bony osteotomy procedures should generally be reserved for children aged 10 years and older. Joint-sparing procedures are preferred over arthrodesis in the pediatric population to preserve joint mobility. Memeo and colleagues reported outcomes of 402 feet treated with arthroereisis at a median follow-up of 130 months, with procedures performed primarily in children within this age range (22). Xu and colleagues treated 15 feet in adolescents with a mean age of 15.2 years using double calcaneal osteotomy, achieving full correction without joint fusion (40). The calcaneo-stop meta-analysis by Galán-Olleros and colleagues, covering 2,394 feet, reported a mean patient age of 11.2 years at surgery (36).

*Level of recommendation: Mild*

#### Recommendation 10. Subtalar arthroereisis as a minimally invasive option

The panel considered subtalar arthroereisis, including the calcaneo-stop technique, as a minimally invasive surgical option for moderate symptomatic flexible flatfoot in children; however, expert consensus was not reached on this recommendation. The available evidence suggests favorable outcomes: a systematic review of 24 studies encompassing 2,550 feet reported excellent patient satisfaction in 79.9% of cases, with an overall complication rate of 7.1% and a reoperation rate of 3.1% (17). A meta-analysis of 17 studies covering 1,536 feet showed that postoperative Meary's angle improved to near-normal at 5.27 degrees and calcaneal pitch increased to 15.70 degrees (35). Hu and colleagues found no significant difference in final AOFAS scores between subtalar arthroereisis and triple C osteotomy, though arthroereisis required shorter operative time and less blood loss (24). Vogt and colleagues compared three arthroereisis methods in 113 procedures and found comparable outcomes but varying implant complication rates: 29% for Kalix, 8% for Giannini, and 3% for SESA devices (25). The divergence in expert opinion likely reflects uncertainty regarding the durability of correction and the heterogeneity of implant-specific outcomes.

Level of recommendation: No consensus* (Delphi agreement: 55%). This label indicates that expert consensus was not achieved at the predefined ≥75% threshold; it does not indicate that the procedure is clinically contraindicated.

#### Recommendation 11. Lateral column lengthening for moderate-to-severe deformity

We recommend lateral calcaneal lengthening osteotomy for moderate-to-severe deformity, particularly when significant forefoot abduction is present, as it achieves greater radiographic correction than arthroereisis. Tahririan and colleagues conducted a randomized clinical trial of 66 patients comparing subtalar arthroereisis with lateral calcaneal lengthening and found that lateral column lengthening achieved somewhat better overall radiographic correction (26). Suh and colleagues confirmed in a systematic review comparing 21 lateral column lengthening studies with 13 arthroereisis studies that lateral column lengthening produced greater improvement in anteroposterior talo-first metatarsal angle and calcaneal pitch (37). However, Moraleda and colleagues reported that calcaneocuboid subluxation occurred in 51.5% of cases after calcaneal lengthening, with an overall complication rate of 18% compared to 10% for triple C osteotomy (23).

*Level of recommendation: Mild*

#### Recommendation 12. Combined and tailored surgical approaches for severe deformity

The panel considered combined and tailored surgical approaches for severe flexible flatfoot with hindfoot valgus greater than 15 degrees, forefoot abduction, and residual forefoot supination; however, expert consensus was not reached on this recommendation. The available evidence supports the use of multiplanar correction in selected cases: Xu and colleagues demonstrated that double calcaneal osteotomy (medializing plus Evans) with or without Cotton osteotomy achieved significant improvement in AOFAS and SF-36 scores in 15 feet of adolescents, preserving joint mobility without fusion (40). Liu and colleagues treated 47 feet in 28 children aged 9 to 14 using tailored combinations of medializing calcaneal osteotomy (89.4%), lateral column lengthening (34%), and Cotton osteotomy (38.3%) with soft tissue procedures, improving the mean AOFAS score from 65.0 to 91.9 (41). Li and colleagues confirmed the value of combining arthroereisis with medial soft tissue tightening for forefoot abduction correction in severe cases (39). The lack of consensus reflects the absence of comparative studies and uncertainty regarding indications for multiplanar vs. single-procedure correction.

Level of recommendation: No consensus* (Delphi agreement: 65%). This label indicates that expert consensus was not achieved at the predefined ≥75% threshold; it does not indicate that the approach is clinically contraindicated.

#### Recommendation 13. Role of soft tissue procedures as adjuncts

We recommend gastrocnemius recession or Achilles tendon lengthening when equinus contracture is present, defined as ankle dorsiflexion less than 10 degrees with the knee extended. For severe deformities, medial soft tissue tightening, including spring ligament repair and talonavicular capsule plication, may be required as an adjunct to arthroereisis or osteotomy to adequately correct forefoot abduction. Additional soft tissue procedures are not recommended for mild-to-moderate deformities already corrected by osteotomy alone. Mosca, in a narrative review, identified equinus contracture as the primary pathological subtype of flexible flatfoot associated with symptoms (6). Li and colleagues showed that adding spring ligament repair and talonavicular capsule plication to subtalar arthroereisis significantly improved forefoot abduction correction in severe deformities (39). However, Moraleda and colleagues found that additional soft tissue procedures did not improve clinical or radiographic outcomes in mild-to-moderate deformities already corrected by osteotomy alone (23).

*Level of recommendation: Mild*

#### Recommendation 14. Outcome assessment and standardized measures

We recommend that treatment outcomes be evaluated using a standardized combination of validated patient-reported outcome measures (AOFAS score, Foot Function Index, Visual Analogue Scale for pain, and PODCI for pediatric function), radiographic parameters (Meary's angle, calcaneal pitch, talonavicular coverage angle, and anteroposterior talocalcaneal angle), and pedobarography where available as a radiation-free adjunct. Heterogeneity in outcome measures is a well-documented limitation in the pediatric flatfoot literature (11, 38). Follow-up should include assessment at a minimum of 6 months, 1 year, and 2 years post-intervention, with at least 4 years of follow-up recommended for surgical patients to detect late recurrence (26, 38). The EPOS survey established that clinical assessment, radiographic measurement, and patient-reported outcomes should be evaluated in combination (11).

*Level of recommendation: High*

#### Recommendation 15. Need for high-quality evidence and registry data

We recommend that future research prioritize multicenter prospective trials with standardized diagnostic criteria, validated outcome measures, and minimum 5-year follow-up, and that multicenter registries be established to accumulate sufficient data for evidence-based guideline refinement. The Cochrane review noted that the evidence for both conservative and surgical treatment of pediatric flatfoot is of low to very low certainty (15). Smith and colleagues found that all 24 studies in their systematic review of subtalar arthroereisis were Level III or IV, with no randomized controlled trials comparing surgical techniques (17). Smolle and colleagues similarly highlighted that no study with a minimum 4-year follow-up exceeded Level IV evidence (38). The EPOS survey documented wide practice variation among 140 specialist respondents across 23 countries, reinforcing the need for standardized consensus (11).

*Level of recommendation: High*

## Discussion

Conservative management should be considered the foundation of treatment for pediatric flexible flatfoot across all age groups. Yet, there is little information on the optimal dose, duration, and type of conservative intervention, and what constitutes an adequate trial of nonoperative care before surgical referral remains poorly defined. These 15 recommendation statements, of which 13 achieved the predefined agreement threshold, represent the first structured attempt by the CPAM-LRC to bridge this knowledge gap through a combination of systematic evidence review and expert polling.

Over the past two decades, the prevailing approach to pediatric flatfoot has shifted considerably. The routine prescription of corrective shoes and rigid insoles for all children with flat feet, once widespread, has given way to a more selective strategy that reserves active intervention for symptomatic patients (1, 6, 15). This evolution reflects accumulating evidence that the medial longitudinal arch develops spontaneously in most children during the first decade and that orthoses do not alter this natural trajectory (5, 15, 32). Observation with parental reassurance remains the mainstream approach for asymptomatic children throughout the first decade of life, with structured clinical reassessment recommended at age 8 to 10 years to determine whether active intervention is warranted. When equinus contracture, persistent pain beyond age 10, or progressive deformity is present, clinicians should proceed from observation to active conservative management (Recommendations 3–7) and, if conservative treatment fails after a minimum 6-month trial, consider surgical evaluation (Recommendations 8–13).

Rehabilitative exercise programs, including gastrocnemius-soleus stretching and intrinsic foot muscle strengthening, can be beneficial as first-line active treatment, particularly given the favorable normalization rates reported by Riccio and colleagues (29). Kinesio Taping as an adjunct to UCBL orthoses may enhance compliance and reduce pressure-related complications in athletic children (31). However, evidence supporting these adjuncts remains limited to single-center studies with short follow-up. The cost of custom orthoses, the time commitment required for structured exercise programs, and the opportunity cost of prolonged conservative management in children with progressive deformity probably outweigh any marginal benefits in cases where surgical indications are already clear. Clinicians should weigh these factors carefully on an individual basis.

A summary of all 15 recommendation statements with their supporting evidence and Delphi consensus ratings can be viewed in Table 3.

The evidence underpinning these recommendations is predominantly of low to very low certainty. The 2022 Cochrane review rated the available evidence for foot orthoses in pediatric flat feet as low certainty across all outcomes (15). Smith and colleagues found that all 24 studies in their systematic review of subtalar arthroereisis were Level III or IV, with no randomized controlled trials comparing surgical techniques head-to-head (17). Despite the low certainty of evidence, expert clinicians participating in the Delphi process were largely in agreement on the core recommendations, particularly regarding the primacy of conservative management, the 6-month minimum trial before surgical referral, and the age-dependent selection of surgical technique.

One area of relative disagreement was the role of custom vs. prefabricated foot orthoses. While the Cochrane review found no advantage for custom devices over prefabricated alternatives in routine flexible flatfoot (15), some panelists expressed concern that this finding may not generalize to children with atypical foot morphology or comorbid conditions such as juvenile idiopathic arthritis. This apparent inconsistency likely reflects heterogeneity in the populations studied and the difficulty of applying trial-level evidence to individual clinical decisions. Recommendation 5 endorses prefabricated orthoses as the default, reserving custom devices for specific clinical subgroups, and emphasizes that all orthotic prescription should target symptomatic relief rather than structural correction.

The term “flatfoot” is often reported interchangeably with “pes planus,” “pes planovalgus,” and “pronated foot” in the published literature, creating confusion across studies and clinical settings. We chose to use “flexible flatfoot” throughout these guidelines to better distinguish this common, reducible deformity from rigid flatfoot secondary to tarsal coalition or other structural pathology. This distinction carries direct therapeutic implications: flexible flatfoot is amenable to conservative management and, when necessary, joint-sparing surgical procedures, whereas rigid flatfoot typically requires a fundamentally different surgical approach (6, 13). Furthermore, clinical assessment and radiographic evaluation should be combined in every diagnostic workup; one cannot replace the other. Physical examination identifies flexibility, equinus contracture, and symptomatic status, while weight-bearing radiographs quantify deformity severity and guide surgical planning (10, 11).

For children undergoing surgical correction, key milestones include pain-free weight-bearing and return to unrestricted physical activity, including sports participation. However, the entire postoperative rehabilitation protocol should be based on progression criteria rather than fixed timelines. Time since surgery is necessary but not sufficient for clearance; radiographic consolidation, clinical range of motion, and functional performance must all be assessed before advancing activity levels. These criteria improve patient protection but remain incompletely validated in the pediatric flatfoot population, and prospective studies are needed to establish their predictive value.

Parental anxiety, particularly concern about long-term disability and the perceived need for intervention, is a significant contributor to overtreatment in pediatric flatfoot (42). This psychological dimension may drive unnecessary orthosis prescription and premature surgical referral. Clinicians should address parental expectations directly, providing clear education about the natural history and the distinction between cosmetic appearance and functional impairment.

Completion of postoperative rehabilitation and clearance to return to physical education is not the same as return to full competitive sport. Children and adolescents who have undergone osteotomy-based reconstruction require a transition phase with progressive exposure to high-impact loading, typically spanning 3 to 6 months after formal clearance (40). This graduated approach is particularly important for adolescent athletes, in whom premature return to sport may compromise correction durability.

The cost of or access to specialized pediatric orthopedic consultation, custom orthotic fabrication, and structured rehabilitation programs might be challenging, particularly in resource-limited settings and rural regions. Where specialist services are unavailable, primary care providers can apply these guidelines using prefabricated orthoses and home-based exercise programs with periodic reassessment at 3-month intervals (18, 30).

Adherence to conservative treatment programs remains a substantial barrier. Clinicians should set expectations early, explaining that conservative management represents a sustained commitment over at least 6 months rather than a quick resolution. Realistic goal-setting is essential: the objective is symptom control and functional improvement, not necessarily cosmetic normalization of the arch. Defining clear milestones, such as pain-free walking and return to sport, helps maintain patient and family engagement. Periodic reassessment every 3 months allows timely identification of treatment failure and prevents unnecessarily prolonged conservative management in surgical candidates.

Resource constraints also warrant acknowledgment. Pedobarography, while recommended as a radiation-free monitoring tool (25), may be unavailable outside tertiary centers. Clinical photography and standardized physical examination scoring systems represent lower-cost alternatives. Advanced imaging modalities such as weight-bearing CT, increasingly used in adult flatfoot research, remain largely inaccessible in routine pediatric practice and are not required for the majority of clinical decisions addressed by these guidelines.

Moving forward, future research should evaluate the implementation impact of these recommendations on practice variation and patient outcomes, clarify objective progression criteria for both conservative and surgical pathways, and improve understanding of the dose-response relationship between orthosis wear time and symptomatic benefit.

### Limitations

Several strengths and weaknesses of this guideline warrant candid acknowledgment. The systematic literature review was comprehensive, spanning multiple databases and encompassing both conservative and surgical interventions across all relevant study designs. The Delphi process involved a multidisciplinary panel of pediatric orthopedic surgeons, podiatrists, orthotists, and methodology experts, strengthening the clinical relevance and methodological rigor of the consensus. However, the inclusion scope was limited to English-language publications, potentially excluding relevant data from other regions. Publication bias assessment was constrained by the predominance of small, single-center studies without prospective registration. Certainty of evidence was assessed at the outcome level using the GRADE framework and at the individual study level using the OCEBM classification, while strength of recommendation was determined independently by Delphi consensus; the scarcity of Level I and II studies meant that most recommendations rested on expert consensus rather than high-certainty evidence. The strength-of-recommendation terminology adopted in this guideline (“High,” “Moderate,” “Mild,” and “No consensus”) is a custom classification based on Delphi agreement thresholds and does not correspond directly to the “strong” vs. “conditional” terminology used in GRADE-based guidelines. Panel composition, while multidisciplinary, was drawn primarily from the CPAM-LRC membership, and recommendations may reflect practice patterns more prevalent in this network. Notably, the expert panel did not include physiotherapists or physical rehabilitation specialists, which is a limitation given that several recommendations address rehabilitative exercise programs and conservative management strategies. Future guideline updates should include these disciplines to ensure broader clinical representation. Patient and caregiver input was not formally incorporated into the Delphi process, a limitation that future updates should address through structured stakeholder engagement and the inclusion of patient-reported priority outcomes.

### Conclusion

This guideline provides 15 recommendations informed by available evidence and expert consensus for the treatment of pediatric flexible flatfoot, encompassing observation, conservative management, and surgical intervention. Observation with parental reassurance remains the recommended approach for asymptomatic children, with structured clinical reassessment at age 8 to 10 years. When symptoms are present, structured conservative management including rehabilitative exercises and foot orthoses for symptomatic relief should be attempted for a minimum of 6 months before considering surgical referral. Subtalar arthroereisis and calcaneal osteotomy represent the principal surgical options, with procedure selection guided by deformity severity and patient age. While the certainty of evidence was low to very low for most components, all 15 recommendation statements were formulated through structured Delphi consensus with 13 achieving the predefined agreement threshold. Future multicenter prospective trials with standardized outcomes and minimum 5-year follow-up are urgently needed to strengthen the evidence base.
