# Supplementary material for: Treatment of pediatric flatfoot: a systematic review-based consensus and guidelines by CPAM-LRC
Source: Front Pediatr. 2026 May 8;14:1825355. doi: 10.3389/fped.2026.1825355 (PMC13194422; doi:10.3389/fped.2026.1825355)
Supplement: Supplementary file 1 [file Table1.docx]

Supplementary Table 1 Search Strategy

Search Date: December 10, 2025

| **PubMed** | |
| --- | --- |
| #1 flatfoot* OR flat foot OR flat feet OR flatfeet OR pes planus OR planovalgus OR flexible flatfoot OR flexible OR hypermobile OR "Flatfoot"[Mesh] | |
| #2 pes planus OR flatfoot* OR planovalgus | |
| #3 pediatric* OR paediatric* OR child* OR infant* OR adolescen* OR toddler* OR schoolchild* OR teen* OR "Child"[Mesh] OR "Infant"[Mesh] OR "Adolescent"[Mesh] OR "Pediatrics"[Mesh] | |
| #4 #1 AND #2 AND #3 | **1986** |
|  | |
| **Web of science** | |
| #1 TS=(flatfoot* OR "flat foot" OR "flat feet" OR flatfeet OR "pes planus" OR planovalgus OR "flexible flatfoot" OR flexible OR hypermobile) | |
| #2 TS=("pes planus" OR flatfoot* OR planovalgus) | |
| #3 TS=(pediatric* OR paediatric* OR child* OR infant* OR adolescen* OR toddler* OR schoolchild* OR teen*) | |
| #4 #1 AND #2 AND #3 | **1900** |
|  | |
| **Embase** | |
| #1(flatfoot* OR "flat foot" OR "flat feet" OR flatfeet OR "pes planus" OR planovalgus OR "flexible flatfoot" OR flexible OR hypermobile):ti,ab,kw | |
| #2("pes planus" OR flatfoot* OR planovalgus):ti,ab,kw | |
| #3(pediatric* OR paediatric* OR child* OR infant* OR adolescen* OR toddler* OR schoolchild* OR teen*):ti,ab,kw | |
| #4 'child'/exp | |
| #5 'infant'/exp | |
| #6 'adolescent'/exp | |
| #7 'pediatrics'/exp | |
| #8 pediatric*:ti,ab,kw OR paediatric*:ti,ab,kw OR child*:ti,ab,kw OR infant*:ti,ab,kw OR adolescent*:ti,ab,kw OR toddler*:ti,ab,kw OR schoolchild*:ti,ab,kw OR teen*:ti,ab,kw | |
| #9 #1 OR #2 | |
| #10 #4 OR #5 OR #6 OR #7 OR #8 | |
| #11 #3 AND #9 AND #10 | **1767** |
|  | |
| **Cochrane** | |
| #1 (flatfoot OR "flat foot" OR "flat feet" OR flatfeet OR "pes planus" OR planovalgus OR "flexible flatfoot" OR flexible OR hypermobile):ti,ab,kw | |
| #2 (pes planus OR flatfoot* OR planovalgus):ti,ab,kw | |
| #3 (pediatric* OR paediatric* OR child* OR infant* OR adolescen* OR toddler* OR schoolchild* OR teen*):ti,ab,kw | |
| #4 MeSH descriptor: [Flatfoot] explode all trees | |
| #5 MeSH descriptor: [Child] explode all trees | |
| #6 MeSH descriptor: [Infant] explode all trees | |
| #7 MeSH descriptor: [Adolescent] explode all trees | |
| #8 MeSH descriptor: [Pediatrics] explode all trees | |
| #9 #1 OR #4 | |
| #10 #3 OR #5 OR #6 OR #7 OR #8 | |
| #11 #9 AND #2 AND #10 | **116** |
