# Supplementary material for: Treatment of pediatric flatfoot: a systematic review-based consensus and guidelines by CPAM-LRC
Source: Front Pediatr. 2026 May 8;14:1825355. doi: 10.3389/fped.2026.1825355 (PMC13194422; doi:10.3389/fped.2026.1825355)
Supplement: Supplementary file 2 [file Table2.docx]

Supplementary Table 2 Basic Characteristics Table

| Title | Comparison of the Influence of Supportiveand Sensorimotor Insoles on Flat Feet in Children- a Double-Blind, Prospective, Randomized,Controlled Trial | Flexible Juvenile Flat Foot Surgical Correction: A Comparison Between Two Techniques After Ten Years’ Experience | Comparison of the Calcaneo-Cuboid-Cuneifom Osteotomies and the Calcaneal Lengthening Osteotomy in the Surgical Treatment of Symptomatic Flexible Flatfoo | A retrospective cohort study comparing the therapeutic efficacy  of three surgical interventions for pediatric flexible flatfoot | Subtalar Arthroereisis for Flexible Flatfoot in Children—Clinical, Radiographic and Pedobarographic Outcome Comparing Three Different Methods | Functional and radiographic comparison of subtalar arthroereisis and lateral calcaneal lengthening in the surgical treatment of flexible flatfoot in children |
| --- | --- | --- | --- | --- | --- | --- |
| Number | 1 | 2 | 3 | 4 | 5 | 6 |
| First Author | Kira-Henriette Liebau | Antonio Memeo | Luis Moraleda | Xiongke Hu | Bjoern Vogt | MohammadAli Tahririan |
| Study Type | RCT | Comparative Study | Retrospective Comparative Study | Retrospective Cohort Study | Single-Center Cohort Study | RCT |
| Intervention/Control | Supportive, sensorimotor insoles vs placebo insoles | Surgery vs surgery | Surgery vs surgery | Surgery vs surgery vs surgery | Surgery vs surgery vs surgery | Surgery vs surgery |
| Year | 2023 | 2018 | 2012 | 2025 | 2021 | 2021 |
| Country | Germany | Italy | Not report | China | Germany | Iran |
| Included Population | Children with flexible flatfoot | Only patients with symptomatic, flexible, idiopathic grade 3–4 flat feet were included, while congenital, neurologic, and posttraumatic flat feet were excluded. | The medical records were carefully reviewed to look for patients with idiopathic flexible flatfoot who had either an Evans’ procedure or a triple Costeotomy. | Children with symptomatic flexible flatfoot aged 6–18 years, failed ≥6 months conservative therapy | Children with Flexible flatfoot (FFF) | Children with flexible flatfoot |
| Intervention Group Age | 6-16years old | 8-16 years old | 11.2±3 years old | 10.6 ± 1.6 years (SESA) | 10.2 (6-14) | 10.06 ± 1.68 |
| Intervention Group 2 Age | 6-16years old | / | / | 10 ± 1.6 years (HyProCure) | 10.3 (5-16) | / |
| Control Group Age | 6-16years old | 8-16 years old | 11.6±2.5 years old | 11.7 ± 3.6 years (Triple Costeotomy group) | 12 (8-16) | 10.19 ± 1.54 |
| Intervention Group Female/Male | Women 4/men 17 | Women 99/men 103 | / | Group distribution not detailed | Women 5/men 6 | Women 12/men 23 |
| Intervention Group 2 Female/Male | Women 5/men 16 | NA | / | Group distribution not detailed | Women 13/men 24 | / |
| Control Group Female/Male | Women 1/men 9 | Women 122/men 78 | / | Group distribution not detailed | Women 10/men 15 | Women 12/men 19 |
| Intervention | Sensorimotor insoles | Exosinotarsal arthroereisis with metallic screw | Calcaneo-Cuboid-Cuneifom Osteotomies (Triple C) | Subtalar extra-articular screw arthroereisis (SESA) | The non-absorbable Kalix® sinus tarsi endorthesis (Kalix®) | Subtalar Arthroereisis (SA) |
| Intervention 2 | Supportive insoles | NA |  | HyProCure implantation at tarsal sinus (HyProCure) | The absorbable Giannini sinus tarsi endorthesis (Giannini) |  |
| Additional Intervention | Foot training | Half plasters at 90° were positioned after surgery for 2 weeks or for 3 weeks if tendon lengthening was performed; after removal of the plaster, walking with crutches (partial weight bearing) was recommended for a week. | / | For those with Achilles tendon contracture, Achilles tendon lengthening was performed. |  |  |
| Control Group | Placebo insoles | Endosinotarsal with bioabsorbable devices | Calcaneal Lengthening Osteotomy (Evans) | Calcaneo-cuboid-cuneiform (Triple C) osteotomy | Subtalar extraarticular screw arthroereises (SESA) | Lateral Calcaneal Lengthening (LCL) |
| Intervention Duration | / | / | / | SESA 31.3±6.3 min，HyProCure 29.2±11. min，Triple C 93.1±15.8min | Kalix: 28.8 months; Giannini: 28.8months; SESA: 2 years; | / |
| Follow-up Duration | 6 months、12 months | 130 months (35-150 months) | Triple C：2.7 ± 2.2 years Evans:5.3 ± 4 years | At least 32 months, average 41 months | 29.0 months (1–111 month) | 17.6 months (11-20 months) |
| Intervention Dose | / | / | / | / | / | / |
| Additional Intervention Group | Once a day | Two weeks after the surgery, a splint will be used, and then a crutch will be needed in another week | Kidner procedure (13)；Medial reefing (6)；tendo-Achilles lengthening(29)；Peroneous brevis lengthening (19)； | For those with Achilles tendon contracture, Achilles tendon lengthening was performed | / | Gastrosoleus Recession |
| Additional Control | Once a day | Two weeks after the surgery, a splint will be used, and then a crutch will be needed in another week | Kidner procedure (10)；Medial reefing (7)；tendo-Achilles lengthening (11)；Peroneous brevis lengthening (6)；Medial cuneiform osteotomy (6)；Calcaneo-cuboidf ixation (12) | For those with Achilles tendon contracture, Achilles tendon lengthening was performed | / | Gastrosoleus Recession |
| Intervention Frequency | / | Single surgery | Single surgery | Single surgery | / | / |
| Total Sample Size | 52 | 402 | 63 | 31 patients (49 feet) | 73 (113 feet) | 66 |
| Intervention Group Sample Size | 21 | 202 | 30 (21) | SESA (10 feet) | 11 (21 feet) | 35 |
| Intervention Group 2 Sample Size | 21 | / | / | HyProCure (21 feet) | 37 (56 feet) | / |
| Control Group Sample Size | 10 | 200 | 33（21） | Triple C（18 feet） | 25(36 feet) | 31 |
| Intervention Group Baseline Value | Height (m)1.29±0.13；weight (KG)26.56±7.73;BMI15.26±1.94;shoe size32.55+3.8;malleolar valgus index32.43±3.72 | 1.CB (Costa Bertani angle)：136.7°; 2.HI (heel inclination angle):13.0°; 3.TDA (talar declination angle):34.6°; 4.KI (kite angle):28.3° | 1.AP Talus-First Metatarsal Angle:21.8 ± 9.3°; 2.Talonavicular Coverage :41 ± 9.2%; 3.Lateral Talocalcaneal Angle:49.1 ± 8.6°; 4.Calcaneal Pitch:11.1 ± 5.4°; 5.Talocalcaneal Index:79.1 ± 17.7 | AOFAS：44.5±6 | Etiology (idiopathic/ neuromuscular):3/8;Side (right/left) (bilateral):10/11 (10);Body mass index in kg/m2 (range):20.5 (16.3–25.0);Lengthening of gastrocnemius muscle or Achilles tendon (yes/no):14/7 | 1.AP Talus-1st Metatarsal Angle (°):30.97 ± 2.80;2.Lateral Talus-1st Metatarsal Angle (°):28.06 ± 2.22;3.Calcaneal Pitch (°):5.71 ± 1.23;4.AOFAS Score:67.28 ± 6.01;5.VAS Pain Score:5.83 ± 0.71 |
| Intervention Group 2 Baseline Value | Height（m）1.40±0.15；weight（KG)34.99±11.30;BMI16.29±4.60;shoe size35.76±3.4;malleolar valgus index32.65±3.99 | / | / | AOFAS：45.8±5.5 | Etiology (idiopathic/ neuromuscular):26/11;Side (right/left) (bilateral):27/29 (19);Body mass index in kg/m2 (range):20.9 (18.5–24.2);Lengthening of gastrocnemius muscle or Achilles tendon (yes/no):22/34 | / |
| Control Group Baseline Value | Height(m)1.35±0.18；weight(KG)32.56±11.83;BMI17.24±2.97;shoe size34.30±3.90;malleolar valgus index31.98±2.75 | 1.CB( Costa Bertani angle）：136.6°; 2.HI(heel inclination angle):12.9°°; 3.TDA(talar declination angle):34.9°; 4.KI(kite angle):28.1° | 1.AP Talus-First Metatarsal Angle:19.3 ± 8.6°; 2.Talonavicular Coverage :38.6 ± 9.9%; 3.Lateral Talocalcaneal Angle:48.7 ± 7.2°; 4.Calcaneal Pitch:11.8 ± 5.2°; 5.Talocalcaneal Index:80.6 ± 10.6 | AOFAS：42.9±6.7 | Etiology (idiopathic/ neuromuscular):19/6;Side (right/left) (bilateral):15/21 (11) ;Body mass index in kg/m2 (range):19.9 (15.4–24.8);Lengthening of gastrocnemius muscle or Achilles tendon (yes/no):15/21 | 1.AP Talus-1st Metatarsal Angle (°):30.45 ± 2.69;2.Lateral Talus-1st Metatarsal Angle (°):28.71 ± 2.41;3.Calcaneal Pitch (°):6.16 ± 1.13;4.AOFAS Score:68.71 ± 5.70;5.VAS Pain Score:5.68 ± 0.75 |
| Funding Reported | Not reported | Not reported | Not reported | Not reported | This research received no external funding. | Not reported |
| Top 3 Adverse Event Types |  | 1.Incomplete Correction：Group A (C-STOP): 23 (11.4%) ； 2. Inflammatory Process：Group B (ENDO):20 (10%)； 3.Screw Issues Requiring Removal：Group A (C-STOP): 9 (4.5%)，Group B (ENDO): 5 (2.5%) | 1.Triple C: Wound dehiscence(1);Tibial stress fracture(1) 2.Evans:Calcaneocuboid Subluxation(17);Pseudoarthrosis of the calcaneal osteotomy（1）；Neuroapraxia of the popliteal portion of the sciatic nerve(1);Pain and stiffness of the subtalar joint(1);Hypoesthesia at the level of the scar(2);Delayed wound healing(1);Complaint of feeling a hole at the iliac crest | 1.Wound pain(Triple C)：16.7%；2.Tarsal sinus pain(HyProCure)：14.3%；3.Tarsal sinus pain(SESA)：10%；4.Fibular muscle contracture(SESA)：10% | 1.Secondary dislocation or breakage:Kalix® (6/21);Giannini(10/56);SESA (0/36);2.Peroneal muscle contractures:SESA(4/36);3.Treatment-related pain due to implant complications:Kalix® (4/21);Giannini(4/56) | 1.Graft Displacement (Control Group - LCL): Occurrence: 1 case (in a female patient), identified 1 month post-op, causing pain 2.Persistent Pain Requiring Implant Removal (Intervention Group - SA): Occurrence: 1 case, complaining of persistent pain 7 months post-op |

Table 2 Basic Characteristics Table

| Title | The Effects of Talus Control Foot Orthoses in Children with Flexible Flatfoot | The long-term use of foot orthoses affects walking kinematics and kinetics of children with flexible flat feet: A randomized controlled trial | Rehabilitative treatment in flexible flatfoot: a perspective cohort study | Short-term effects of customized arch support insoles on symptomatic flexible flatfoot in children A randomized controlled trial | Treating symptomatic flexible flatfoot deformities. a novel  technique: comparison of uc berkeley laboratory foot orthosis  with and without kinesio taping in juvenil athletes |
| --- | --- | --- | --- | --- | --- |
| Number | 7 | 8 | 9 | 10 | 11 |
| First Author | So Young Ahn | AmirAli Jafarnezhadgero | Ilaria Riccio | Ru-Lan Hsieh | Cem Sever |
| Study Type | RCT | RCT | Prospective cohort study | RCT | Retrospective Comparative Study |
| Intervention/Control | Orthosis vs orthosis | Orthosis vs insole | Rehabilitation therapy vs orthotic insoles | Insoles vs no treatment | Foot orthoses + taping vs foot orthoses |
| Year | 2017 | 2018 | 2009 | 2015 | 2024 |
| Country | Korea | Iran, Germany | Italy | Taiwan | Türkiye |
| Included Population | Children aged >6 years diagnosed with flexible flatfoot | Boys aged 8-12 years with flexible flat feet | Children with a diagnosis of flexible flatfoot | Children with symptomatic flexible flatfoot, age 3–10 years | Patients with SFPP |
| Intervention Group Age | 9.59 ± 4.24 years | 10.5 ± 1.4 years | 3.4 ± 0.55 | 6.9 ± 0.6 years | 9.2±2.9 |
| Intervention Group 2 Age | 10.14 ± 4.99 years | 10.4 ± 1.5 years | 2.6 ± 0.52 | 6.2 ± 0.4 years | 8.2±1.7 |
| Control Group Age | 10.14 ± 4.99 years | 10.4 ± 1.5 years | 2.6 ± 0.52 | 6.2 ± 0.4 years | 8.2±1.7 |
| Intervention Group Female/Male | 8/12 | 0/15 | 116/184 | 12/14 | 14/13 |
| Intervention Group 2 Female/Male |  |  |  |  |  |
| Control Group Female/Male | 8/12 | 0/15 | 118/219 | 12/14 | 10/13 |
| Intervention | Talus control foot orthosis (TCFO) | Medial arch support foot orthoses (custom-made) | Rehabilitation / therapeutic exercise program (including flexibility, strengthening, sensory–proprioceptive training, etc.) | Customized arch support insoles (thermoplastic, medial longitudinal arch support) | UCBL foot orthosis plus kinesio taping (KT) |
| Intervention 2 |  |  |  |  |  |
| Additional Intervention | Wear instruction and gait training | Progressive wearing time from 1 hour to full day (On the first day, application time was restricted to one hour to allow familiarization. On every following day, application time was increased by one hour until participants finally wore the FOs (EG) / insoles (CG) for the full day) | Caregivers (usually mothers) learned and delivered exercises at home; periodic PT follow-ups; small-group physiotherapy sessions | 1 participant had additional medial heel wedge (0.4 cm); 1 had medial forefoot and heel wedges | KT as an adjunct/co-intervention to the foot orthosis |
| Control Group | Rigid foot orthosis (RFO) | Flat 2-mm-thick insoles (sham) | Historical control: insoles and orthopaedic footwear/orthoses | No insoles | UCBL foot orthosis alone (no KT) |
| Intervention Duration | 12 months | 4 months | Intervention group: mean 2.75 years (min 2.1, max 4.3) | 12 weeks | Mean follow-up: 28.6 ± 4.3 (26) months |
| Follow-up Duration | >8 hours per day | Average daily wearing time: 6.8 ± 3.8 hours (EG), 7.0 ± 3.7 hours (CG) | (1) First month: 30 min/session; (2) then: at least 2 h/week home exercises; (3) monthly: additional 30-min small-group PT | At least 5 hours/day | UCBL wear time: at least 8 hours per day (both groups) |
| Intervention Dose | / | / | / | / | / |
| Additional Intervention Group |  |  |  |  |  |
| Additional Control |  |  |  |  |  |
| Intervention Frequency | Daily | Daily | First month: 3×/week; then ≥2 h/week (reported as time/week); monthly 1 additional 30-min small-group PT | Daily | KT cycle: worn for 3 days; no tape on day 4; re-applied after a 1-day interval |
| Total Sample Size | 40 | 30 | 637 | 52 | 50 |
| Intervention Group Sample Size | 20 | 15 | 300 | 26 | 27 |
| Intervention Group 2 Sample Size |  |  |  |  |  |
| Control Group Sample Size | 20 | 15 | 337 | 26 | 23 |
| Intervention Group Baseline Value | Age(years)9.59±4.24;Sex, M:F (No.)12:8;Height (cm)139.28±12.78;Weight (kg)37.41±11.33;BMI19.18±2.39 | Age(years) 10.5 ± 1.4；Body height(cm)142.4 ± 5.7；Body mass(kg)48.1 ± 9.1；BMI20.0±4.0；Navicular drop(mm)13.0±2.1；AHI 0.18±0.07；Calcaneal eversion(degree)7.2±1.1 | No. of children = 300; No. of feet = 600; Age (years, mean ± SD) = 3.4 ± 0.55; Male (%) = 61.3; Treatment period (years, mean ± SD) = 2.75 ± 0.63; Grading (%) = I 0; II 35.7; III 64.3 | Age: 6.9±0.6 y; Beighton: 7.2±1.9; Navicular drop: 12.7±4.2 mm; Foot posture index: 9.0±1.9; Lat calcaneal inclination: 15.0±5.0°; Lat calcaneal-1st metatarsal: 13.7±6.8°; AP Talo navicular: 18.2±6.7° | Age (years)：9.2±2.9；Baseline AOFAS (intervention): 58.00 ± 5.83 |
| Intervention Group 2 Baseline Value |  |  |  |  |  |
| Control Group Baseline Value | Age 10.14±4.99；Sex, M:F (No.)12:8;Height (cm)138.23±10.17;Weight (kg)35.13±16.93; BMI 18.37±4.67 | Age(years) 10.4 ± 1.5；Body height(cm)141.2 ± 6.1；Body mass(kg)48.2 ± 9.4；BMI20.1±4.2；Navicular drop(mm)13.1±1.9；AHI0.18±0.06；Calcaneal eversion(degree)7.1±0.9 | No. of children = 337; No. of feet = 674; Age (years, mean ± SD) = 2.6 ± 0.52; Male (%) = 64.99; Treatment period (years, mean ± SD) = 2.66 ± 0.61; Grading (%) = I 0; II 41.2; III 58.8 | Age: 6.2±0.4 y; Beighton: 7.7±1.2; Navicular drop: 14.2±2.4 mm; Foot posture index: 9.6±1.9; Lat calcaneal inclination: 16.1±5.2°; Lat calcaneal-1st metatarsal: 11.5±6.7°; AP Talo navicular: 19.0±8.1° | Age (years)：8.2±1.7；Baseline AOFAS (control): 56.00 ± 5.69 |
| Funding Reported | None reported | Deutsche Forschungsgemeinschaft (DFG), Open Access Publishing Fund of University of Potsdam | No funding statement found (only COI statement present) | Supported by grants from Shin Kong Wu Ho-Su Memorial Hospital and Ministry of Science and Technology, Taiwan | Funding: Open access funding provided by TÜBİTAK; no funding source for the study |
| Top 3 Adverse Event Types | Not reported | Not reported | Not reported | Not reported | Adverse events : (1) pressure sores (2) superficial dermabrasion (3) tape-related allergic reactions |
