# Supplementary material for: Treatment of pediatric flatfoot: a systematic review-based consensus and guidelines by CPAM-LRC
Source: Front Pediatr. 2026 May 8;14:1825355. doi: 10.3389/fped.2026.1825355 (PMC13194422; doi:10.3389/fped.2026.1825355)
Supplement: Supplementary file 3 [file Table3.docx]

| **Number** | **First Author and Year** | **1. Randomization** | **2. Allocate hidden** | **3. Subject-Operator blind method** | **4. Outcome-Blind method** | **5. Missing data** | **6. Selective Reporting** | **7. Other** | **Grade** |
| --- | --- | --- | --- | --- | --- | --- | --- | --- | --- |
| 1 | Kira-Henriette Liebau et al.2023 | Low risk | Unclear | Low risk | Low risk | Low risk | Low risk | Low risk | Moderate risk |
| 6 | Mohammad Ali Tahririan et al. 2021 | Low risk | Low risk | Low risk | Low risk | Unclear | Low risk | Low risk | Moderate risk |
| 7 | Ahn et al, 2017 | Low risk | Unclear | High risk | Unclear | Low risk | Low risk | Low risk | High risk |
| 8 | Jafarnezhadgero et al. 2018 | Low risk | Low risk | Low risk | High risk | Unclear | Low risk | Low risk | High risk |
| 10 | Hsieh et al. 2015 | Low risk | Low risk | High risk | Unclear | Low risk | Low risk | High risk | High risk |

**Supplementary Table 3 Quality Assessment of Randomized Controlled Trials Based on Randomized Organized Bias**
