# Supplementary material for: Treatment of pediatric flatfoot: a systematic review-based consensus and guidelines by CPAM-LRC
Source: Front Pediatr. 2026 May 8;14:1825355. doi: 10.3389/fped.2026.1825355 (PMC13194422; doi:10.3389/fped.2026.1825355)
Supplement: Supplementary file 4 [file Table4.docx]

**Supplementary Table 4 Outcome Assessment Based on GRADE**

| **Number** | **Included in the study** | **Intervention measures** | **Outcome** | **Bias risk** | **Inconsistency** | **intermittent** | **Inaccuracy** | **Publication bias** | **Quality** |
| --- | --- | --- | --- | --- | --- | --- | --- | --- | --- |
| 1 | Kira-Henriette Liebau et al.2023 | sensorimotor insoles/supportive insoles | 1.MVC | Moderate | No report | Low | Low | No report | Low |
|  |  |  | 2.valgus inde | Moderate | No report | Low | Low | No report | Low |
|  |  |  | 3.FADI | Moderate | No report | Low | Low | No report | Low |
|  |  |  | 4.the static contact area | Moderate | No report | Low | Low | No report | Low |
|  |  |  | 5.the dynamic contact area | Moderate | No report | Low | Low | No report | Low |
| 2 | XAntonio Memeo et al.2018 | A-STOP vs ENDO | 1.pain scale | High | No report | Low | Low | No report | Moderate |
|  |  |  | 2.CB | High | No report | Low | Low | No report | Moderate |
|  |  |  | 3.HI | High | No report | Low | Low | No report | Moderate |
|  |  |  | 4.TDA | High | No report | Low | Low | No report | Moderate |
|  |  |  | 5.KI | High | No report | Low | Low | No report | Moderate |
| 3 | Luis Moraleda et al.2012 | Triple C vs Evans | 1.AP talo-first MTT angle（Measured on Anteroposterior and Lateral Weight-bearing Radiographs） | High | No report | Low | Low | No report | Moderate |
|  |  |  | 2.AP talocalcaneal angle（Measured on Anteroposterior and Lateral Weight-bearing Radiographs） | High | No report | Low | Low | No report | Moderate |
|  |  |  | 3.Talonavicular coverage（Measured on Anteroposterior and Lateral Weight-bearing Radiographs） | High | No report | Low | Low | No report | Moderate |
|  |  |  | 4.AP talo-fifth MTT angle（Measured on Anteroposterior and Lateral Weight-bearing Radiographs） | High | No report | Low | Low | No report | Moderate |
|  |  |  | 5.L talo-first MTT angle（Measured on Anteroposterior and Lateral Weight-bearing Radiographs） | High | No report | Low | Low | No report | Moderate |
|  |  |  | 6.L talocalcaneal angle（Measured on Anteroposterior and Lateral Weight-bearing Radiographs） | High | No report | Low | Low | No report | Moderate |
|  |  |  | 7.Talohorizontal angle（Measured on Anteroposterior and Lateral Weight-bearing Radiographs） | High | No report | Low | Low | No report | Moderate |
|  |  |  | 8.Calcaneal pitch（Measured on Anteroposterior and Lateral Weight-bearing Radiographs） | High | No report | Low | Low | No report | Moderate |
|  |  |  | 9.L calcaneo-fifth MTT angle（Measured on Anteroposterior and Lateral Weight-bearing Radiographs） | High | No report | Low | Low | No report | Moderate |
|  |  |  | 10.L first-fifth MTT angle（Measured on Anteroposterior and Lateral Weight-bearing Radiographs） | High | No report | Low | Low | No report | Moderate |
|  |  |  | 11.Naviculocuboid overlap (%)（Measured on Anteroposterior and Lateral Weight-bearing Radiographs） | High | No report | Low | Low | No report | Moderate |
|  |  |  | 12.Talocalcaneal index（Measured on Anteroposterior and Lateral Weight-bearing Radiographs） | High | No report | Low | Low | No report | Moderate |
| 4 | Xiongke Hu et al.2025 | SESA vs HyProCure vs Triple C | 1.AP talo-first MTT angle（Radiologic parameter） | High | No report | Low | Low | No report | Moderate |
|  |  |  | 2.AP talocalcaneal angle（Radiologic parameter） | High | No report | Low | Low | No report | Moderate |
|  |  |  | 3.Talonavicular coverage（Radiologic parameter） | High | No report | Low | Low | No report | Moderate |
|  |  |  | 4.L talo-first MTT angle（Radiologic parameter） | High | No report | Low | Low | No report | Moderate |
|  |  |  | 5.Calcaneal pitch（Radiologic parameter） | High | No report | Low | Low | No report | Moderate |
|  |  |  | 6.L talocalcaneal angle（Radiologic parameter） | High | No report | Low | Low | No report | Moderate |
| 5 | Bjoern Vogt et al.2021 | Kalix® vs Giannini | Treatment satisfaction. | High | No report | Low | Low | No report | High |
| 6 | Mohammad Ali Tahririan et al. 2021 | SA vs LCL | 1.AP Talus-1st Metatarsal Angle (°) | High | No report | Low | low | No report | Moderate |
|  |  |  | 2.Lateral Talus-1st Metatarsal Angle (°) | High | No report | Low | low | No report | Moderate |
|  |  |  | 3.Calcaneal Pitch (°); | High | No report | Low | low | No report | Moderate |
|  |  |  | 4.AOFAS Score | High | No report | Low | low | No report | Moderate |
|  |  |  | 5.VAS Pain Score | High | No report | Low | low | No report | Moderate |
| 7 | Ahn et al, 2017 | TCFO vs RFO | 1.APTCA | Moderate | Low | Low | High | No report | Low |
|  |  |  | 2.RCSP | Moderate | Low | Low | High | No report | Low |
|  |  |  | 3.CP | Moderate | Low | Low | High | No report | Low |
|  |  |  | 4.LTTCA, LTTMA | Moderate | Low | Low | High | No report | Low |
| 8 | Jafarnezhadgero et al. 2018 | Medial arch support foot orthoses vs sham insoles | Kinematics (ankle, knee, hip angles) | Low | Low | Low | Low | Low | High |
|  |  |  | Kinetics (GRF) | Low | Low | Low | Low | Low | High |
| 9 | Ilaria Riccio et al. 2009 | Rehabilitation therapy vs orthopedic insoles | Improvement of the arch of the foot shape | High | No report | High | Moderate | No report | Very low |
| 10 | Hsieh et al. 2015 | Customized arch support insoles | 1. Patient-reported outcome: Pain/comfort (PODCI) | Moderate | LOW | LOW | High | LOW | LOW |
|  |  |  | 2. Patient-reported outcome: Transfer and basic mobility (PODCI) | Moderate | LOW | LOW | High | LOW | LOW |
|  |  |  | 3. Patient-reported outcome: Upper extremity and physical function (PODCI) | Moderate | LOW | LOW | High | LOW | LOW |
|  |  |  | 4. Patient-reported outcome: Sports and physical function (PODCI) | Moderate | LOW | LOW | High | LOW | LOW |
|  |  |  | 5. Patient-reported outcome: Global function (PODCI) | Moderate | LOW | LOW | High | LOW | LOW |
|  |  |  | 6. Patient-reported outcome: Happiness (PODCI) | Moderate | LOW | LOW | High | LOW | LOW |
|  |  |  | 7. Patient-reported outcome: Physical Health Summary Score (PedsQL) | Moderate | LOW | LOW | High | LOW | LOW |
|  |  |  | 8. Patient-reported outcome: Psychosocial Health Summary Score (PedsQL) | Moderate | LOW | LOW | High | LOW | LOW |
|  |  |  | 9. Patient-reported outcome: Total Score (PedsQL) | Moderate | LOW | LOW | High | LOW | LOW |
|  |  |  | 10. Performance-based outcome: Stair ascent time | Moderate | LOW | LOW | High | LOW | LOW |
|  |  |  | 11. Performance-based outcome: 10-m fast walking time | Moderate | LOW | LOW | High | LOW | LOW |
|  |  |  | 12. Performance-based outcome: 10-m normal walking time | Moderate | LOW | LOW | High | LOW | LOW |
|  |  |  | 13. Performance-based outcome: Stair descent time | Moderate | LOW | LOW | High | LOW | LOW |
|  |  |  | 14. Performance-based outcome: Timed Up and Go | Moderate | LOW | LOW | High | LOW | LOW |
|  |  |  | 15. Performance-based outcome: Chair rise time | Moderate | LOW | LOW | High | LOW | LOW |
| 11 | Cem Severet al. 2024 | UCBL +KT vs UCBL alone | 1.AOFAS | Serious | No report | High | High | No report | Very low |
|  |  |  | 2.Radiographic measures | High | No report | High | High | No report | Very low |
|  |  |  | 3.Adverse events | High | No report | Moderate | High | No report | Low |

**Note：**MVC (maximum voluntary contraction)；FADI（Foot and Ankle Disability Index）；CB (Costa Bertani angle)；HI (heel inclination angle)；TDA (talar declination angle); KI (kite angle); A-STOP（exosinotarsal arthroereisis with metalic AO screws）:ENDO( endosinotarsal correction with bioabsorbable device); Triple C (Calcaneo-Cuboid-Cuneiform Osteotomies); Evans (Calcaneal Lengthening Osteotomy）; SESA(Subtalar extra-articular screw arthroereisis ); HyProCure(HyProCure implantation at tarsal sinus); Kalix® (the non-absorbable Kalix® sinus tarsi endorthesis);Giannini (the absorbable Giannini sinus tarsi endorthesis); SA(Subtalar Arthroereisis）;LCL(Lateral Calcaneal Lengthening）; APTCA(anteroposterior talocalcaneal angles); RCSP (resting calcaneal stance position); CP(calcaneal pitch); LTTCAs(lateral talocalcaneal angles), and LTTMAs(lateral talometatarsal angles); UCBL(University of Cali fornia Berkeley Laboratory); KT(kinesio taping )
