# Supplementary material for: Treatment of pediatric flatfoot: a systematic review-based consensus and guidelines by CPAM-LRC
Source: Front Pediatr. 2026 May 8;14:1825355. doi: 10.3389/fped.2026.1825355 (PMC13194422; doi:10.3389/fped.2026.1825355)
Supplement: Supplementary file 5 [file Table5.docx]

**Supplementary Table 5 Quality Assessment of Cohort Studies Based on NOS**

| **Number** | **Title** | **1**）Representativeness of the Exposure Cohort | **2)Selection of non-exposed cohorts** | **3)Confirmation of Exposure** | **4)No outcome events occurred for the research subject prior to the commencement of the study.** | **5**）Comparability of cohorts derived from design or analysis | **6**） Evaluation of the Concluding Event | **7**） Is the follow-up period sufficient to observe the occurrence of the outcome? (e.g., 5 years) a) Yes (a sufficient follow-up period was selected to observe the occurrence of the outcome) * b) No | **8)**Completeness of follow-up | **Score** |
| --- | --- | --- | --- | --- | --- | --- | --- | --- | --- | --- |
| 2 | Flexible Juvenile Flat Foot Surgical Correction: A Comparison Between Two Techniques After Ten Years’ Experience | **c** | **a*** | **a*** | **a*** | **Not reaching a** | **a*** | **a*** | **c** | 5 |
| 3 | Comparison of the Calcaneo-Cuboid-Cuneiform Osteotomies and the Calcaneal Lengthening Osteotomy in the Surgical Treatment of Symptomatic Flexible Flatfoo | **d** | **c** | **a*** | **a*** | **Not reaching a** | **a*** | **b** | **d** | 3 |
| 4 | A retrospective cohort study comparing the therapeutic efficacy  of three surgical interventions for pediatric flexible flatfoot | **b** | **a*** | **a*** | **a*** | **a*** | **a*** | **a*** | **d** | 6 |
| 5 | Subtalar Arthroereisis for Flexible Flatfoot in Children—Clinical, Radiographic and Pedobarographic Outcome Comparing Three Different Methods | **b** | **a*** | **a*** | **a*** | **Not reaching a** | **a*** | **b** | **d** | 4 |
| 9 | Rehabilitative treatment in flexible flatfoot: a perspective cohort study | **b** | **a** | **a** | **a** | **a + **** | **d** | **a** | **d** | 2 |
| 11 | Treating symptomatic flexible flatfoot deformities. a novel  technique: comparison of uc berkeley laboratory foot orthosis  with and without kinesio taping in juvenil athletes | **c** | **a*** | **a*** | **a*** | **a*** | **a*** | **a*** | **b*** | 7 |
